# Collective trauma in the Vanni- a qualitative inquiry into the mental health of the internally displaced due to the civil war in Sri Lanka

**DOI:** 10.1186/1752-4458-4-22

**Published:** 2010-07-28

**Authors:** Daya Somasundaram

**Affiliations:** 1 Department of Psychiatry, University of Jaffna, Sri Lanka

## Abstract

**Background:**

From January to May, 2009, a population of 300,000 in the Vanni, northern Sri Lanka underwent multiple displacements, deaths, injuries, deprivation of water, food, medical care and other basic needs caught between the shelling and bombings of the state forces and the LTTE which forcefully recruited men, women and children to fight on the frontlines and held the rest hostage. This study explores the long term psychosocial and mental health consequences of exposure to massive, existential trauma.

**Methods:**

This paper is a qualitative inquiry into the psychosocial situation of the Vanni displaced and their ethnography using narratives and observations obtained through participant observation; in depth interviews; key informant, family and extended family interviews; and focus groups using a prescribed, semi structured open ended questionnaire.

**Results:**

The narratives, drawings, letters and poems as well as data from observations, key informant interviews, extended family and focus group discussions show considerable impact at the family and community. The family and community relationships, networks, processes and structures are destroyed. There develops collective symptoms of despair, passivity, silence, loss of values and ethical mores, amotivation, dependency on external assistance, but also resilience and post-traumatic growth.

**Conclusions:**

Considering the severity of family and community level adverse effects and implication for resettlement, rehabilitation, and development programmes; interventions for healing of memories, psychosocial regeneration of the family and community structures and processes are essential.

## Background

*Tham Thimithimithom ThaiyathomTham ThimithimithomLiving we were- on Vanni soilLiving we wereEducating ourselves we were - JoyfullyEducating ourselves we wereRunning around we were - with friendsRunning around we were*

*Came the airplanes- on usThrowing bombsDied relations- ourRelations fellRace destroyed- TamilRace disappeared*

*Life destroyed- ourLife scatteredSuffering saw- weSadness imposedCaged by war- we wereTrapped in sufferingEnough the sorrow- weEscape to survive*

Song/Poem by Vanni IDP school student

What happened in the Vanni and to its people from August 2006 onwards, particularly from January 2009 to May 2009, has been described in apocalyptic (in the local Tamil as *pralayam*) terms[1-4]. The total destruction of civilian infrastructure that ensued in the bitter fight to the end between the Sri Lankan military forces and the Liberation Tigers of Tamil Eelam (LTTE) with an estimated civilian population of around 300, 000 trapped in between is an ineffable human calamity. A common refrain from people who were there has been '*varthayal varnicca mudiyathavai *(it is beyond description by words)'[5]. When one meets or sees survivors even in January, 2010 in the various internment camps, public places like bus stands or in private homes, they are obviously in a *thihaiththupona (*daze) state, not having comprehended or come to terms with what happened. They stand out from the rest of humanity. Much of what happened is still shrouded in mystery and secrecy. There are several contested versions, discourses battling to establish their perspective. The Sri Lankan state and military have actively striven to suppress the truth of the ensuing carnage for fear of investigations for war crimes [4,6-8]. There also appears to be a more long term effort to frame and reconstruct the collective memories and historical record in line with the political agendas of different actors. The Lankan state and Sinhala nationalist would like to paint it as a war against terrorism, deny an ethnic or minority problem and portray the Tamils as of relatively recent origin, migrants or invaders from South India in the last millennium [9-11]. Indeed, internationally the LTTE had become branded as a terrorist organization by several countries including India, U.S., U.K., Canada, European Union, Australia, Malaysia and others. In contrast, Tamil nationalists depict the conflict as a liberation struggle of a suppressed minority, claiming the Tamils have inhabited the North and East from the beginning of history [12-14]. However, the psychosocial and mental health impact on the civilian population and the interventions for their recovery remains a major concern addressed by this qualitative study.

Since the work of Sigmund Freud, it has become a basic principal aim of psychotherapy to bring out the repressed memories and associated emotions as a process of healing. This cathartic effect is believed to help people come to terms with what has happened and carry on with their lives. Following massive ethnic conflicts in South Africa, Rwanda and Bosnia there were attempts at reconciliation through 'healing of memories' using techniques like truth commissions. If people can be given an opportunity to express their stories through words, poems, songs, drama, drawings or other creative arts, it is believed that would help in their recovery. It would provide some meaning for the enormous suffering they have undergone, hope for the future and trust in the world. It would also help others understand what has happened as well as create an enabling atmosphere for resolving contrasting views.

Memories can change over time depending on internal and external conditions. This is always a challenge in psychoanalysis and narrative ethnography. Child abuse, trauma, depression, grief, fear, wishes, desires and other strong emotions can repress or distort memories. Similarly, the external political environment or socio-cultural milieu can determine what can and what cannot be said. Silence in a situation of 'repressive ecology'[15] is a survival strategy that can become ingrained and permanent. Thus peoples' memories can become a field of intense contest, memories can be erased, and others created or changed. This paper will attempt to give a voice, narrate the stories, access the memories and describe the lived experience of those caught up in the fateful Vanni episodes from different perspectives as a psychosocial method of catharsis, a healing of memories.

Complex situations that follow war and natural disasters have a psychosocial impact on not only the individual but also on the family, community and society. Just as the mental health effects on the individual psyche can result in non pathological distress as well as a variety of psychiatric disorders like Post Traumatic Stress Disorder (PTSD); massive and widespread trauma and loss can impact on family and social processes causing changes at the family, community and societal levels. A better understanding of the supra-individual reality can be sought through the ecological model of Bronfenbrenner [16] with the micro, meso, exo and macro systems or the individual nested in the family nested in the community [17,18]. Previous workers had already drawn attention to the community level problems caused by disasters. Kai Erikson [19] gives a graphic account of ***Collective Trauma ***as '**lo*ss of communality' ***following the Buffalo Creek disaster in the US. He and colleagues described the 'broken cultures' in North American Indians and 'destruction of the entire fabric of their culture' due to the forced displacements and dispossession from traditional lands into reservations, separations, massacres, loss of their way of life, relationships and spiritual beliefs [20]. Similar tearing of the 'social fabric' has been described in Australian aboriginal populations [21]. There was a description of '***cultural bereavement****' *due to the loss of cultural traditions and rituals in Indochinese refugees in the US [22] and collective trauma due to the chronic effects of war[23]. More recently, a number of discerning workers in the field have been drawing attention to the importance of looking at the family[24-27] and cultural dimension[28-31] following disasters. Finally, Abramowitz [32] has given a moving picture of '***collective trauma' ***in six Guinean communities exposed to war.

### Context

**Figure 1:**
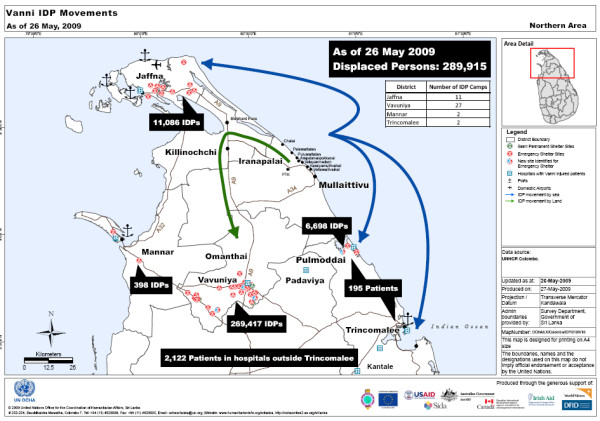
**Map of Vanni IDP's **[72]. Movement of the Vanni IDP's

The area called the Vanni compromises mainly the Districts of Killinochi and Mullaithivu and adjoining parts of Vavuniya and Mannar Districts in Northern Sri Lanka (see map- fig. 1). With the more recent migrations, an estimate of the total population would have been between 300,000 to 400,000 consisting exclusively of Tamils. Due to conflicting political compulsions the exact number remains controversial [2].

### Folk lore, myth and history

The hoary beginnings of the people of the Vanni (*Vanniar*) ruled over by chieftains called Vannians [33,34] has not been clearly established but settlements have been dated back 2000 years[35]. There is mention in the *Konesar *culvet and old *vya *song of sixty *Vanniar *coming from Madurai in South India accompanying the royal bride for the king at Anuradhapura in the first century BC [36]. They were settled in *Adangapattu *(Unsuppressed place)[37] while one became a Dishava in Kandy. Interestingly for long periods from the 1990's the whole of the Vanni together with other areas in northeast under LTTE control were called 'uncleared' (meaning not under state control) areas by the state. Adangapattu district is again mentioned as the residence of Paranda Vanniyan in Colonial British accounts. Vanni came into the historical limelight around the beginning of the eleventh century [33,34,38,39] when the Cholas from South India exerted their influence over Jaffna and encouraged settlements in the Vanni. And later, more prominently in the thirteenth century, the political space for the Vanni opened up to assert itself when according to partisan versions, 'invasions by (South) Indian mercenaries', Magha from 'Kalinga' the most notorious of them, were blamed for the fragmentation of the Anuradhapura and Pollanaruwa Kingdoms of the Rajarata civilization [40]. These ethnocentric, somewhat mythic, accounts of the past feed into present day ethnic emotions, consciousness, polarized perceptions, relations and conflict[41,42]. However, other, more scholarly accounts, ascribe the breakdown to internal dynastic power struggles [43,44] and the neglect of the hydraulic infrastructure of the ancient civilization and consequent breeding of pernicious malarial Anopheles mosquitoes [45]. The malaria ridden forests of the Vanni functioned as a buffer zone between the North and the South and could have been one of the primary causes for the separate evolution of the two ethnic identities. The Vanni chieftains appear to have paid some tributary to the more powerful rulers in the north or south as the power balance happened to be at that time, but had an independent spirit with a distinct *naddar *culture [46,47] and dialect (language) of their own. Dissident and defiant groups found safe haven in the impenetrable forests of the Vanni from where they mounted reprisal attacks. However, this original group of peoples, way of life and language have now been assimilated into the mainstream cultures. Historically, the Vanni encompassed Mannar, Vavuniya, Trincomalee, Pollannaruwa, Batticaloa, Ampara and Puttalam hinterlands [33]. The name Vanni is said to be derived from the Sanskrit and Tamil word for forest (*vannam*) or fire (*vahni*) , but there is also some historical evidence in Culverts and old songs that the Vanniar could have originally come from the large Vanniar clan/caste from North Arcot in South India [33]. One of the traditional old temples is at Vattapallai dedicated to *Kannahi *or *Pattini deyo *in Sinhala. It is from here that an annual pilgrimage (*paddayattarai*) goes along the coast and then through forests to Kattirgamam (Kattragamma in Sinhala) in the South East. In the western, Mannar side of the Vanni, Thirkatheeswaram temple and the Catholic Christian Madhu church, built on an old Amman temple, are popular places of pilgrimage of 'Hindu' Tamils, 'Buddhist' Sinhalese, 'Christians' and others.

The Vanniar are also reputed to belong to the warrior caste with heroic and marital skills. According to folklore, seven Vanni chieftains who fought unsuccessfully against the Dutch committed suicide to avoid capture. They are still revered as heroic *devas *(gods) at *Natchimar *temples in the Vanni and Jaffna where lamps will be lit and drums beaten in their names every Tuesday and Friday [Ahalankan, unpublished manuscript]. The most famous of the Vanni chieftains was *Pandara Vanniyan *or *Wanni Bandara *in Sinhalese, the last king of the Vanni who fought against the Dutch and British colonial powers [48,49]. In alliance with the Kandy kingdom he drove Lt. von Drieberg and his garrison from the Mullaithivu fort capturing their canons and 'overran the whole of the northern districts (Vanni) and the boldness to penetrate as far as Elephant pass into the Jaffna Peninsula'[50]. From conventional warfare, he resorted to guerilla attacks and was finally defeated by Lt. von Drieberg when the British organized a three pronged attack from Jaffna, Mannar and Trincomalee around 1803. This was followed by 'burning of all his houses and his people were dispersed into the jungle, and eventually out of the Vanni. The power of the Vanni Chiefs was thus finally and effectually extinguished' [50]. Interestingly, folklore has it that Lt. von Drieberg was originally with the Dutch forces where he felt humiliated by Pandara Vanniyan for having defeated him several times, including in personal combat, and had been permitted to withdraw. He had stayed on after the Dutch were ousted by the British to fight on to defeat Pandara Vanniyan. The similarity to Gen. Sarath Fonseka who developed a passionate zeal to defeat the LTTE and Prabhakaran after being trapped in the early 1990's at Pompamadu near Chettikulum in the Vanni by the LTTE when a Lt. Colonel and later, surviving a near fatal suicide attack is striking. He led the war in the Vanni and was responsible for systematically and relentlessly pursuing the LTTE till they were completely destroyed. He became a Sinhala national hero of epic proportions but ironically, with the twist of power politics, he is to be court martialed for treason for revealing evidence of war crimes [51]. Pandara Vanniyan was declared a national hero by the prime minister and a statue of him was opened in 1982 with much fanfare in Vavuniya at the main junction where the A-9 Highway between Jaffna and Kandy (and Colombo) meets the road to Mannar (and further down the road to Trincomalee) [49]. More recently, the LTTE leader, Prabhakaran, has been compared to him by present day Tamil nationalists, Karunanidhi the current Chief Minister of Tamil Nadu, India in his book, *Payum Puli Pandara Vanniyan*, and Nedumaran. The historical parallels to what happened in the Vanni recently are remarkable except ordinary civilians were not used as hostages.

The old village, agricultural settlements of the Vanniar were mainly centered around water resources such as tanks and ponds outside the present Vavuniya town called Villangkulam earlier. The villages were reputed for their cooperative activities and absence of much caste or class distinctions or conflict. The settlements were mainly Tamil except in the north east and south eastern parts of Vavuniya there were a mixture of Sinhala and Tamil families while on the western side there were Muslim and Tamils, all of whom lived peacefully together. Vavuniya town developed with the opening of road and railway connection by the British between Jaffna in the North and Kandy and Colombo in the South. Those who came on official duties or traders settled in the town. The town grew to its present size after it became a border town with the LTTE controlled areas to the north, a centre of trade and goods moving north and later haven for refugees from other areas.

Killinochchi and Mullaithivu districts were sparsely populated, jungle areas with agricultural settlements around tanks like Iranaimadu and some permanent but largely migrant (from the western coast during their southwest monsoon) fishing villages on the Eastern coast. During the 1970's there were concerted efforts to settle unemployed, educated youth in the Vanni and involve them in agriculture and animal husbandry. Following the state acquisition of British owned estates in 1974 resulting in starvation on the estates [52] and the 1977, 1983 anti-Tamil pogroms, Tamils from the south and hill country settled in increasing numbers in the Vanni. With the Lankan operation *Riviresa *to retake the Jaffna peninsula, the LTTE engineered the 1995 exodus from Jaffna which saw around 200,000 people with the LTTE moving to the Vanni [53]. If the people had been cornered with the LTTE in Jaffna there may have been a high number of civilian casualties then [54] as happened later in the Vanni in 2009. With the 2002-6 peace accord, some of these people moved back to their original homes, several of whom were targeted by state-affiliated killer squads after the resumption of war in 2006.

The LTTE leadership and cadres faced annihilation when they took on the Indian army in the form of Indian Peace Keeping Force (IPKF) in 1987 in Jaffna [55]. Eventually they had to withdraw into the Vanni and into the Mullaithivu jungles. Several efforts by the IPKF to round up the LTTE leadership culminated in an operation called 'Check mate' using the famed Gurkha regiment to go into the Mullaithivu jungles [56] where they cornered the LTTE, but were not permitted to proceed by Indian politics. The Indian generals complained they had to fight with one hand tied behind their back. Prabhakaran had appealed to Karunanidhi, with a personal letter addressing him as the only hope, the star of the Tamils. In the 2009 final battle too, the LTTE had pinned considerable hope on Tamil Nadu politics. Karunanidhi the chief minister in Tamil Nadu had gone on a publicity fast but called it off when the Lankan state promised not to use heavy weapons and offered a ceasefire. Some of the narrative accounts mentioned people listening intently on the radio amidst the raging battle for news of the election results from India that came in just before the last onslaught, dashing all hopes.

However, at that time the LTTE was still using guerilla tactics using civilians as shields and contrived civilian casualties [55]. With the withdrawal of the IPKF in 1990, the LTTE gradually consolidated their hold in the Vanni and gradually changed from a guerilla force into a conventional army holding onto territory. They had some spectacular military successes in expelling the Lankan state forces from several garrison military complexes in the Vanni, particularly Mankulam, Killinochchi, Mullaithivu, Pooneryn, and Elephant Pass inflicting enormous casualties and capturing heavy weapons. Over the years, they managed to stave off several attempts by the Lankan state forces to retake or even create in roads into the Vanni. Killinochchi changed hands several times and a concerted operation ('Jayasikuru'- victory assured) to bisect the Vanni along the A9 highway was beaten back by counter attacks called 'unceasing waves' by the LTTE. Nevertheless, the Lankan state held onto the Southeastern Vanni renamed Weli-oya from the Tamil name, Manal aru in 1984 by expelling the Tamil population and creating garrison settlements [57]. This successful policy may foreshadow what may now be attempted for the rest of the Vanni.

With the consolidation of their military control over the Vanni, the LTTE gradually built up an alternate administrative structure in the Vanni amounting to an autonomous, separate de facto state [12]. There were separate police, judicial, financial (tax, bank), administrative, medical, social and other services. When the major A9 was opened up after the 2002 peace accord, there were tight custom, immigration and emigration control at the border crossing points. There was always some form of blockade of goods going into the Vanni by the state, as a result outside goods were always in short supply and cost much higher. Local produce sold at a lower price.

There was a certain atmosphere of Tamil nationalism, a feeling of autonomous independence, a Camelot of sorts- a Tamil de facto state with the illusion of liberation. Tamil language and culture was in unhindered if not exclusive use. The head of the UNICEF programme in the Vanni, an Australian with long experience in Sri Lanka, described the children there as being different from those that she had seen elsewhere in the North East. It was only in the Vanni that children could be seen to play freely, frolicking and jumping into and swimming in the water tanks and irrigation channels. Outside visitors were amazed at the order, organization, sanitation and activity. The Sarvodya leader from the south remarked that in the whole of Sri Lanka it was only in the LTTE controlled areas that women felt safe to walk by themselves late in the night. Unlike in the rest of Sri Lanka, military weapons, check points, barbed wire and round ups were not visible. The 2002-2006 peace period had particularly been specially propitious in this respect. However, the LTTE maintained a fascist, totalitarian control over the civilian population with a network of prisons for dissidents and enemies (*throhies*) [58] who were killed or tortured and a strict pass system that did not allow people under their control to leave the Vanni. They effectively dispelled the whole Muslim population from the North in 1990 and the Sinhala population much earlier. However, the Sinhala state managed to maintain a garrison Sinhala population at *Manalaru *(changed to *Welioya *in Sinhala) in the South East of the Vanni [57]. With the resumption of hostilities in 2006, the A9 highway was closed in August. However, the Lankan forces concentrated first on Eastern Lanka and brought it under their control before moving to retake the Vanni. For the Sri Lankan state there was the historic opportunity to destroy the LTTE once and for all, a designated terrorist organization that had been plaguing the country for a quarter of a century in a long drawn out debilitating civil war situation. They had marshaled all their resources, prepared, planned well from past lessons and apparently garnered international sanction in the post 9/11 '*war against terror*' climate. They attempted to separate the civilians from the LTTE, to coax and pressurize them to leave the fighting areas. However, they would not allow humanitarian concern for civilian casualties get in the way of the chance to finish off the LTTE. From the Lankan state perspective, the Vanni civilians were not exactly innocent: by staying on in the Vanni under LTTE control they had compromised themselves. "*High-level statements have indicated that the ethnic Tamil population trapped in the war zone can be presumed to be siding with the LTTE and treated as combatants, effectively sanctioning unlawful attacks*" [1]. In September, 2008 the state ordered the UN and other international humanitarian agencies to leave the Vanni[59]. They did not allow journalists or independent human rights monitors into the area. Journalists, media and opposition politicians who reported adversely about the state or forces were intimidated, killed or silenced. According to reliable health workers in the field and civilian testimony, the maximum damage, both civilian deaths and injuries, was from the massive, relentless shelling of the civilian population, declared safety zones and hospitals. The Vanni population had already experienced the full brunt of state terror and had all the reasons to be afraid of the advancing army [60,61]. In the recent collective memory would have been the killing of 61 school children and youth in an air raid in Mullaithivu in August, 2006 reported by UNICEF and the Sri Lankan Monitoring Mission (SLMM) [62]. Seventeen aid workers (working for the French International Non Governmental Organisation Action Contre La Faim (ACF) had been executed by the advancing state forces at Muttur in the East [63]. Over 120 civilians seeking refuge at St. Peter's Church in Navaly had been killed by bombing in 1995 [64]. There had been many such massacres of civilians by state forces [65] in the living memory of the Vanni people, some of which they themselves had barely survived. Many had lost a relation or faced the wrath of the forces. An epidemiological survey by a team from the University of Konstanz, Germany using the UCLA PTSD Child Reaction Index with expert validation (Kappa .80) carried out in the Vanni in early 2000's had found that 92% of primary school children had been exposed to potentially terrorizing experiences including combat, shelling, and witnessing the death of loved ones. Twenty five met the criteria for PTSD[66]. There was ever ongoing abductions, torture, disappearances and extrajudicial killings of Tamils by the state forces and the paramilitaries allied with them[67]. For the LTTE as the structures of their de facto state and territory crumbled all around them in face of the State forces' juggernaut, they desperately clung onto the civilians as human shields towards the later stages. They apparently hoped that the unfolding human tragedy would precipitate an international intervention [5]. The LTTE also forcefully recruited men, women and children, gave them increasingly minimum training and pressed them into battle. As a consequence many died and the returning bodies caused increasing friction with the once loyal and passive Vanni civilians. Thus the twin forces of onslaught of the state forces and the LTTE's trapped the civilians. The Vanni population and the Tamils had learned to live between the terror and the counter terror, the parallel authorities and violence of the LTTE and the state [68], but nothing had prepared them for what was to come.

The forces launched well planned, concerted attacks from multiple fronts but the main advance was from the west. As the Lankan forces advanced using heavy artillery shelling and bombing from the air, people fled eastward and then northeastward, through Killinochchi to Mullaithivu to end up in a sliver of land on the East coast. Food became scarce and expensive, there were reported deaths due to starvation, clean water difficult to find, medical help and supplies became non-existent as people fled from one place to another seeking some respite from the continuous shelling and firing. People lay dead on the streets and in their hastily dug bunkers. Some 20,000 to 40,000 are estimated to have died in the apocalyptic carnage [2,8,69,70]. The injured cried for help, while bleeding to death where no one stopped to give a lending hand in their own desperation to escape. The elderly and disabled were left behind. Orphaned children were wandering aimlessly amidst the chaos of blocked roads and desperate humanity. Those who managed to escape this unfolding human tragedy were fired upon by both sides, were injured or killed, had to wade through deep waters, becoming separated and losing all their belongings. Once on the army side, they were checked, some separated and never seen again. They were then herded into buses and taken to temporary shelters and finally interned for months in barbed wire camps for months without access to the outside world [71]. The total thus interned in various Internally Displaced Camps (IDP) camps in Vavuniya, Mannar and Jaffna was just under 300,000 [72] (see Figure 1). The narratives, drawings, poems and interviews presented here are from those interned in these camps and those outside in hospitals and living with friends and relations.

## Methodology

This paper is mainly a qualitative inquiry [73-76] into the psychosocial situation of the Vanni IDP's and their ethnography using narratives and observations obtained through participant observation; in depth interviews; key informant, family and extended family interviews; and focus groups using a prescribed, semi structured open ended questionnaire. Ethical clearance for the study was sought from the Ethical Review Committee of the University of Jaffna. Informed consent was obtained before administration of the questionnaire. Interviews were carried out by the author and by trained psychosocial workers who are involved in assisting the Vanni IDP's. The sampling frame were all those who had lived in the Vanni of northern Sri Lanka and been affected by the outbreak of the war between the state forces and LTTE in the period 2008-9 and eventually displaced as so called IDP's to Vavuniya, Mannar and Jaffna. Generally the sampling has been purposive and convenient such as clinic, hospital patients; displaced and refugee populations; and those accessible living with friends or relations. The transcripts and translations were verified with those involved wherever possible. The author did the translations from Tamil into English for this paper. There were severe limitations to access to IDP camps and to obtaining 'information'. The narratives, drawings, letters and poems as well as data from observations, key informant interviews, extended family, focus group discussions and media reports were analysed for impact at the family and community levels. The key informants included government (Assistant Government Agent (AGA), Gamma Sevakas (GS), Social services, Women affairs, Child Rights and other officers from AGA office, International Non Governmental Organization (INGO), NGO workers, doctors, health staff, Teachers, priests, Camp officers, community leaders (e.g. chairman, president and other members of committees, organizations)- all working with Vanni IDP population. Groups included, camp groups, women groups, extended family groups, community groups (adolescents, religious, mothers, teachers, doctors, health staff). Qualitative analysis of data used standard qualitative techniques like Narrative analysis (content, idioms and structure analysis to locate common epiphanies, contexts, themes, processes, unique features, and semiotics); Phenomenology (personal and family experiences in essence, meaning, experiential description); Grounded theory (selective coding and interrelate categories to develop propositions, conditional matrix, alternate interpretations, themes, hypothesis, and theory); ethnography (cultural, religious and social contexts, events, actors, themes and patterned regularities to interpret how the culture worked in this situation); Case studies (using categorical aggregation to establish themes and patterns, direct interpretation and natural generalizations to extract in-depth picture of cases); and Discourse analysis (read and interrogate the data for patterns, perspectives; historical, mythical and sociopolitical contexts; actions, implications and social reality). The attempt was to '*extract the meaning and implications, to reveal patterns or [and] to stitch together descriptions of events into a coherent narrative' (quoted from Corbin & Strauss 2008)*[72]

The resettlement of the Vanni IDP's is being planned and implemented. The paper pleads for their trauma and psychosocial needs be taken into consideration for their necessary healing and success of rehabilitation and development process. The Tamil community needs these narratives to come out to show the extent of their suffering, for their own review of what has happened and where they are going and for the outside world to understand. For the nation, the eventual process of reconciliation needed for her survival and future progress, the stories of ordinary people has to be told. Social justice, at least steps towards acknowledgement of what has happened would help towards long term psychosocial well being.

The psychosocial phenomena of collective trauma is explored and interventions suggested. the term collective trauma is being introduced to represent the negative impact at the collective level, that is on the social processes, networks, relationships, institutions, functions, dynamics, practices, capital and resources; to the wounding and injury to the social fabric. The long lasting impact at the collective level or some have called it tearing in the social fabric [21] would then result in social transformation [77], of a sociopathic nature that can be called collective trauma. Collective events and consequences may have more significance in collectivistic communities than in individualistic societies [78]. The individual becomes embedded within the family and community so much so that traumatic events are experienced through the larger unit and the impact will also manifest at that level.

## Results

### Narratives

Many ended up in the Vanni after many previous displacements to escape the chronic terror of continuing warfare. The following youthful narrative starts when the person was a young child but is quite typical and shows the complexities:

*As a child we were living in Jaffna when the first major blow in life happened in the 10*^*th *^*month of 1995 with the announcement to leave Valikammam. My friends said that we would be just going today and returning tomorrow. With the clothes I was wearing and two old hand baggage (on foot) we reached the Navatkulli bridge which was rumoured to be broken by nightfall. In our haste, we crossed through mud that reached my neck, lost one of my bags and somehow made it to Chavakachcheri in two days. Here there was the appeal that "Vanni soil will make you live" and some compulsion *(by the LTTE, though not named) *that made us join thousands of people to journey by sea to the Vanni. We experienced two strong emotions during this journey, one was the terror for the navy- when they would cut us up *(people crossing the Killali lagoon were set upon by navy patrols) *and other was the longing when we would return to Jaffna. The nostalgia for Jaffna lasted for days turning into a day dream that continued for years. After this we were displaced again from Killinochchi in 1996. I lost both my parents in 1998. Then my brother was killed in a bomb blast. I came down with malaria several times*. (Health officials reported a high (epidemic) number of deaths due to malaria during that period. But public health measures brought it under control). *I went to school and sat for the national exams from Killinochchi. After that the Killinochchi resettlement process. We gradually became part of the Vanni soil (Vanni man vasihal). The thoughts of Jaffna faded slowly from our minds. Our longing was for freedom. Not necessarily by arms but that we should govern in our land. We wanted rule by the people because our past ethnic leaders had made many historical blunders (varallattu thavaruhal). Whether we liked it or not, we were forced to accept the struggle (porrattam). Although many of our expectations may not come to pass, at least one day, freedom and after that dawn (vidivu). This was the longing of many. Many lost much for this goal. But now we regret that the last 30 years have all been in vain. This anguish is greater than all the suffering we have been through.*

*The 4*^*th *^*phase of the Eelam war resulted in enormous suffering for the people that cannot be described. In 2006 August, the pathway to Jaffna and prize of the peace process, the A-9 highway was closed (puddu villa). Then began the forced conscription with the call, "one person for each house to guard the nation, come forward swiftly (virainthu vareer)". We'll hear loud wailing for the dead (marana olam). When we went to inquire, we would be informed that it was due to forced conscription. It was the oppari *(wailing) *by the conscripts and their relations. I learnt the reason for the wailing later. Many who were taken never returned. This was coerced. Some parents willingly gave their children. Willing or unwilling, some joined because of others. They hoped that somehow a change will come. Subsequently the displacements took hold like a cancer. A common saying became, "we gave our child and eventually we have to leave our home".*

**Figure 2:**
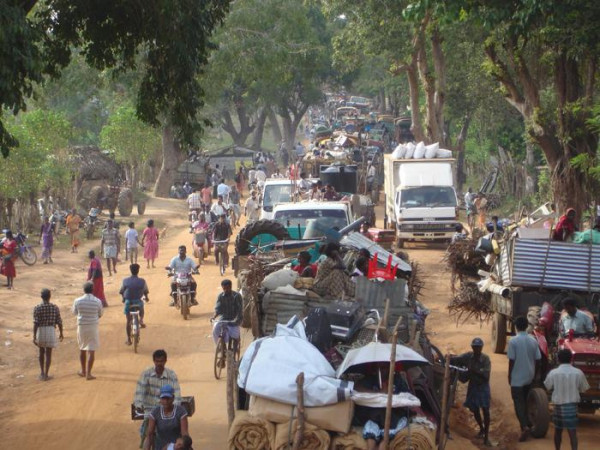
**Displacements **[79].

*Our displacement from Killinochchi started in October, 2008.The reason was that shells from Mallavi and aerial attacks. The planes would drop their bombs somewhere but the pieces would spread to cause damage elsewhere. Among the planes the MIG 29 was a demon. Its sound still rings in my ears. First displacement was to Visvamadu. Everything except the walls of the house were removed. Some even took the bricks that were not cemented. This was due to the bad experiences from the last displacement (on returning they found everything possible to remove had been looted). Our household loaded two 'kandar' (heavy vehicle). Everything from a broomstick were carefully loaded and secured before moving. Somehow we will take everything possible. Then we will return with everything safely was the misplaced belief. Some even uprooted their croton plants to take with them. There was relief, a pleasure in the feeling that we had loaded all our belongings in a heavy vehicle (see Figure *2*: Displacements*[79]*. What happened was different. We were displaced 8 times. For folks from Mannar district it was 16 times. The heavy vehicle finally became by foot (changing from tractor, land master, motor cycle, to bicycle).The items taken became finally one or two handbags, in this the story of those who crossed a waterway to reach (army) control is distressing: some finally even lost their identity card. The first displacement did not appear that major to us. In the belief that they would soon return people said, "the army will come up to Paranthan, after they have all come, they will be chased back by those responsible (the word LTTE was not used). After that we can return, no." Even after our 8*^*th *^*displacement, these were the words of faith used by people. After that they added safe to Visvamadu and declared it the safety zone. Relief was twofold. But it didn't last even 28 days. Attacks towards Visvamadu started. This was the most terrible harvest of the 30 years of war. With it rain floods became frightening. Nature also played with our people. Chickens that people had brought with them were swept away in their cages. Tharappal *(tarpaulin- plastic sheet) *cottages were swept away. Water will seep through the ground of the huts that we built. We became used to these hardships. With these burdens, sweet news reached us of worldwide ahimsa protests by the Diaspora and the neighbouring country's political drama all gave us fresh hope. It was like the person longing for rice receiving buriyani that was sweet only to our ears.*

*There was a strong expectation that India would do something among the Vanni people. At least they would put a stop to the shelling. The reason was the political drama that unfolded in Tamil Nadu in 2008. This appeared to us to be a big change there. After that was the dream of the Indian relief boat. Even in the midst of the horrible war the story spread of the Indian ship coming with food and clothes. We waited two months and spent two days (standing in queues) to finally get the parcel. Before enjoying it, the next displacement came. We had to leave many of the items behind. One thing became clear that people had a strong belief to the last that India would come to their rescue. One could see the sticker from the parcel, "From the Indian people" stuck on the tharapan shacks of the people for a long while afterwards. The next displacement was to Mullaithivu's Vallipunam. We could not stay here safely even for one week. Many shells landed suddenly close by. In the morning people had cooked the chickens they had been carrying as they were becoming tired of carting them around. But before they could eat their meal they had to flee leaving the food behind. With the loud explosions the ground shook. We fell to the ground on top of each other crying, "O God". Many died in that multibarrel attack *(24 to 56 shells are fired almost simultaneously as a single salvo). *There was a young woman very close by with a child bleeding from its mouth. I do not know how to describe the scene. She was leaning onto a tree. When I approached I found there was life. With the neighbours help I had her sent to the hospital on my motorcycle. Afterwards as we were rushing to Thevipuram, which had been declared a safety zone by the state, a child cried, 'brother look somebody's leg is lying there'. I didn't even turn to look as I pushed on in a hurry with an elder on my cycle. People were rushing in all directions not knowing where to go. The next day when I rose my heart was beating fast. As the shelling had subsided, I returned to the earlier place and inquired from those there about the child. They said that on reaching the hospital, the child had survived for just one hour before dying. This had happened in front of my eyes. I had begged God that child should not die. The news of its death caused terror in me. I had comforted many, but could not comfort myself.*

*Severe terror started in Thevipuram. Both sides played firing shells in turn. If you fire ten, I will fire hundred, raining shells. Some of these did not fail to fall on ordinary people. At this stage, many people from Irudumadu and Suthanthirapuram crossed over to army controlled areas. Not easily but amidst great difficulty: "come" they call but continue to shell. "Do not go, stay" and they (LTTE) continued shelling. We also do not want to go. "Our own place, our livelihood, we know the journey *(struggle) *we have already undergone, but who sir, is going to save us? Are we made of steel?" Shells were raining down on us. Parents with the children they have borne. Many obstacles: water comes up to the legs, a child can be carried on the hips; water comes up to the neck, the child can be put on the head; but when the water goes above the head, the mother puts aside the child she has carried so far with great difficulty to try and save herself. People will run... if someone is injured, they would leave the person and continue running. There were parents who left their injured child behind. I saw this with my own eyes at the Mother Mullai church. Again the safety zone became a place of danger. At this point I had to go the Mathanan hospital to send an elderly person by the ICRC ship. For this I had to stay one week at the hospital (now the area from Mathanan to Vaduvahal has been declared the safety zone). While staying at the hospital I came to realize in reality what I had imagined hell to be like. Without a hand, without a leg, bowels protruding out, burnt bodies without any portion left to burn, without eyes and so on of human suffering that one cannot think of. The injured would be brought in continuously from time to time. Of these, those who died on the way to hospital and those dying with or without treatment would be registered at the hospital. Who would take those who had already died due to injuries? Some died as a family. Some bodies would by lying by the side of the road. But I would like to record one thing, the selfless service of the TRO workers who interred the dead bodies to preserve human dignity cannot be forgotten. (*Tamil Rehabilitation Organization a local NGO under the LTTE that did yeoman service for the public [80] but was categorized as part of the 'terrorist' organization by the state*).*

I first learnt of kotu kundu (cluster bombs) in Mathalan. One would hear the click of the shell being loaded but would begin to think there is no explosion, perhaps it is a dud before there would be multiple 'parapara' sounds. Then that area would be mayhem. Not one or two but many would come and fall. In one day, it was not intervals between shelling but their absence would last only for a small time. Most had dug bunkers. Many lived in open bunkers. Some trusted the open skies as their roof. In the last four months, most of our life was spent in bunkers. What has to be noted here is the continuous displacement, people had to move on. With other important things, the logs for the bunkers had also to be carried along. The last place that was declared as the safety zone was bare land used for drying fish. If one dug bunkers there, within one feet there would be water. So many built shelter bunkers above the ground. The seacoast became public toilets. Close by people had to put up their tharrapan shacks and live densely as there was no space. If one attended to their toilet needs in the early morning, they had to be patient till nightfall. Females suffered particularly. Some controlled their urination the whole day before passing it once it became dark. One could observe this directly. Many said they restricted their eating and drinking because of this. Then came the move to Mullivaikal...

*Youths and children with dreams and hopes of life were killed. Conscription of a person for each house changed to whole households being taken for the war effort. Church doors were broken open and my close friend together with other youths (males and females) were conscripted whilst they were praying inside. He was a very spiritual person. I was also on my way to the church in search of succor. The state of the church made me cry, "Is this your fate, the place where people come searching for comfort?" The words of Jesus, "If this is the fate of a green tree, what would become of the dry?" came to mind as I went in search for my friend. I saw the mother's crying face. She could not speak. The family had already sacrificed one member for the war, and now those left had also been conscripted leaving the mother as the lone tree. I learnt later that my friend escaped in two weeks to return to his mother. Words cannot describe the hardships they went through to avoid conscription again. Female and male youth, even children tried many ways to save themselves from conscription. Some hid in holes dug in concealed places. Some hid in jungles. Some died due to shells falling where they hid. But due the continuous displacement they were caught while moving. Some married secretly to save themselves*(there appeared to be a belief that married persons would be spared. Although this was true in earlier times, towards the later stages everyone was taken)*. Some were involved in this forced conscription. Some made themselves leaders. They made their own laws and were the cause of the split from the people. The selfishness of some, those who put their families and their own selves above others became the cause of problem. They stopped us saying the devil *(army) *was out there, but then sent on their own families. People finally asked, "To whom are you showing the devil?"*

*Mullivaikal became very scary. Our environs were hit by multibarrel (40) shells. We did not know what was happening. The surrounding palmyrah were burning. I fell without realization. After a few moments, I look around. Everywhere there was oppari *(wailing). *The elder in the next shack was killed while eating. I had just talked to him. He had said that he had not eaten in the morning *(due to shortage of food), *only at midday. I had seen the 14 year old female child next door cooking a rotti. It was around 12 noon. The shells hit at around 1 PM. The white rice the elder was eating had turned red. One of the rotti's that the child had been cooking was thrown on top of our torn tharappan roof. The child's abdomen had been torn asunder and was eventually sent by ship to Pulmoddai. Deaths became common. Some died inside the bunkers. They would then all be simply buried therein.*

*The World Food Programme *(WFP) *would distribute relief items. We had to stand in queues for it. It would start shelling and we would have to run. Even when dry rations were obtained amidst all these difficulties, there was always shortage. There was floor, sugar, dhal and oil. We became habituated to just Rice and dhal. There was not even an ulli to add. We developed diarrhea and had to go to the toilet often. Shells would come at any time. The price of food items skyrocketed. One coconut was Rs. 1000. Spinach Rs. 150. Once some spoilt carrot and pumpkin came by boat. Unripe mango was Rs. 100 to 150. Some mothers cried for rice...*

*Gunshots also started to target people in Mullivakal. When we looked outside from the bunkers we would see the trees riddled with bullets. Some described as 'dumdum' a type of bullet. An achchi (elderly lady) was sitting by our side when a 'dum' sound was heard. Later she realized her leg was broken. We later realized that a channam (round or bullet) had struck her leg and then exploded again within. Another type of missile was called 'cannon'. These were later fired continuous and many died as a result. One does not hear the 'canon' being fired or know it is coming, only after it has exploded. After this even the thorn bush at Mullivaikal couldn't stand up. Continuous missiles, rockets, gunfire, and with that bombardment from the sea. The bunkers were built facing the sea, to avoid the multipronged attacks from the land. But now shells started coming from the seaside also. For comfort we ran towards Vellammullivaikal. This was only 500 m away. In the middle the night, a hidden arms dump had exploded with burning flames. We are afraid to come out (of bunkers) in the fire light. Vandu *(unmanned aircraft) *are taking pictures from the sky. If people leave the bunkers to come out, at least five shells will come there. Somehow we manage to run towards Vellamullivaikal. On the way we duck for cover, but that turns out to be more frightening than where we had been. There was a school in Vellammullivaikal where the injured were being brought. This was the Vanni hospital. If I am to describe all this it would take a book. However, in short: in the front yard there were many injured. Some were corpses; by the side were the badly injured without anyone to care for them. If it was head injury nobody would even turn to look. There were two or three government doctors. However, trained local doctors *(TEHS) *saved many people*. (Thamil Eelam Health Service was part of the elaborate de facto state infrastructures and institutions evolved by the LTTE. The parallel[68] health service consisted of medical services to the militant cadres that included doctors trained in their medical school, nurses and other medics running frontline first aid centres, field hospitals and base hospitals that carried out complicated surgeries, blood transfusions and rehabilitation[81]. Theelapan Memorial Health Services provided primary health care to civilians throughout areas under their control. Other institutional structures included White Pigeon Artificial limb organization, Centre For Health Care(CHC), Ponnambalam Memorial Hospitals and expatriate visiting specialists).

*This hospital also sustained attacks. The sad part was that the place where people came searching for medicine became their grave. Shells fell on those who were already dead. There was saying that even after death devastation continues became a reality here. Before coming to Vellamullivaikal we celebrated our last mass in the Vanni. Under a tharrapan, the priest and people had done the pusai *(mass) *sitting on the floor (this was my first such experience) as there was no space and gunshots were crossing overhead. The day and time when ICRC ships used to come gave people some respite as shells became less. Yet, some who trusted this and got out died.*

*13/5/2009. World War I remembrance day. I am reading the bible in my bunker while heavy attacks are going on around. I feel my face being covered by mud. Immediately I come out of the bunker and start running. An artillery shells falls nearby covering a bunker with mud. Five children are in the bunker. Thank god they are alive. We dig them out. I turn to look, a woman is squatting on the ground with her head bent. "Iyoo, it's a known girl". I turn her head. She is still alive but there is blood pouring from her nose and ear. Immediately we run from there. 100 m from there we get into another bunker with other known people. A girl who had been talking to us leaves the bunker to come back bent over, holding her abdomen with tears rolling down her eyes. She is immediately taken to the same hospital. There were no vehicles. So two sticks are put through a sarong that functions as the stretcher. She is soon operated on and sent back. She lives with us for three days in the crowded bunker. She would cry for water but we could not give even water because of the abdominal injury. On the third night, while looking at her two children she passed away. The funeral was held inside the bunker. Within three hours we buried her in our hut by the bunker. We also cooked and were eating when an arms dump (store) nearby starts burning. This happens in many places. People grab what they can carry by hand and run where they can. Everywhere it was the same situation. This was the last stage for the Vanni. Those who believed in something became disoriented. Many of these were highly vulnerable innocents. Our faith till the last had been with god. We were very keen to listen to news till the end. Every expected the UN to intervene. NATO will send in troops. Many believed the US statement to the last (*Obama administration had at one stage suggested plans to send US Air Force and Navy units attached to its Pacific Command (PACOM) to evacuate the civilians). *Those concerned *(LTTE) *would surrender their arms to a third party. Civilians will be rescued from the government announced safety zone (up to Vadduvahal) by the intervention of outsiders and taken to a safe place. But the truth was that instead of saving the people the world nations and UN committees respected the sovereignty of a weaker developing country more. But will they avoid intervening needlessly in a sovereign country when their interest is at stake? Laws are for man, man is not restrained by laws. Laws are important but we who were facing death did not have anybody to comfort us at the end. Those who believed till the end kept looking towards the sea for a saviour. The last hope dissolved with the Indian election results. Many did not know what was happening to the end. They just stayed in the bunkers. What has happened to them?*

On the last day we cross the Vaddukaval bridge. Even at the end they (LTTE) block us. But the flood of people had to cross the final blockade. After 30 years of war, more than the changes in the map or the changes in the economics and structure of Illangai (Sri Lanka), who will fill the wounds and trauma in the minds of the people who have suffered these horrors? We, who have learnt to be patient, will wait for peaceful coexistence.

In this case the displacements had started in 1977:

#### Despair

We were originally from Alaveddy in Jaffna. My father was transferred as the post master to Anuradhapura in 1948. We settled in at the post office quarters in new town Anuradhapura. My four younger sisters, one younger brother and myself were born in Anuradhpura. My mother was a good entrepreneur. Through her efforts we saved on our expenses, invested in land, cattle and paddy fields. We built a big house behind St. Joseph's Church and I studied at the Convent in Sinhalese. Talking and talking Sinhala we forgot our Tamil. Our neighbours were Sinhalese, Muslims and Burghers. They were good people. We had no problems. We shared our good and bad times. They helped us a lot. As my mother had many cows and sold milk she was fondly called 'kiriamma' (milk lady) by the Sinhala folks.

The '56 disturbances did not affect us much. The '77 disturbance cannot be forgotten. People who had eaten and drunk from us came with knives, poles and axes to cut us. A few who were grateful to us saved us. We hid for 2 days in the Thisava irrigation canals and jungles without thanni venni (anything). We were sent to Jaffna with police security. With just the clothes we wore we landed at Duraippah stadium in a lorry. Like us there were many others. We stayed at the mission house of the catholic Fathers at Parathithurai. My mother did not like Jaffna. We bought land in Killinochchi and moved there in '77 itself. The refuge life that started in '77 has not ended yet. Unable to live with the Sinhalese we came to Tamil land, but here also it is so. All our property, goats, cattle, chickens, household goods, everything was taken by those around us. My mother will become upset if Sinhalese is spoken, "They have betrayed (irandaham) the house where they ate". Rather than believe in them, we can live in our land, it will be only for a short time..." the lady repeated and passed away crying.

*"If we could not live there in peace, we came to Tamil land but after '85 this has become hell" she said with perumuchu (*deep, sighing breathing*- *A common Tamil cultural idiom of distress [82,83]*.... "When are we going to be able to live in peace?"...*

My sister returned to Anuradhapura but I stayed on in Killinochchi with my children. The children would go to my sisters' at Anuradhapura during school holidays. My second daughter was 8 years old. She had gone to my sisters' for the March holidays in '85. The palapona (decadent) iyyakam movement (LTTE) one day shot many Sinhala folks. If they are shot would they just wait? They hunted down Tamils. Ours somehow reached the army camp and sought refuge there. A soldier who went berserk (irathaveri) started shooting killing and injuring many Tamils. In that my innocent daughter was also killed. I did not even get to see her body. She was buried there. From that day I have not stopped crying thinking of her.

*In '90 my 3*^*rd *^*son joined the movement. From that day my nimmathi (contentment) also went. Day and night, I begged God that nothing would happen to him. Palapona God also had no compassion, he took him in 2000. In 2001 my brothers son had gone to Vavuniya. He died in a claymore that had been set for someone else. We were displaced repeatedly living in sheds. When peace comes we would come back to our own place. Like this we have experienced untold difficulties and tortures to live with the land. In 2002 with the peace agreement , we repaired our home, hoping to life with contentment when the palapona war started again in 2008. Artillery did not let us live in peace. The beatings of the heart from the sound of Kifir drives us into bunkers. The army had come out of Mannar to reach Akarayan. We were not able to stand the shelling and Kifir attacks, the children removed our roof, doors, windows and everything and moved to Visuvamadu. We did not live there with contentment for even 2 months. Leaving most of our goods, we moved to Vallipunam but the Sinhalese did not let us be there for even 2 weeks. With whatever they could get, the children made shelters. They took us to Iranaipallai. Like that we changed places again and again to finally reach Mullivaikal. It was there that I was injured in my arm by a multibarrel shell. My children sent me by ICRC ship (to Trincomalee). My children experienced all types of difficulties to walk from Mullivaikal to Vadduvan to reach the army. They were in Menik farm for 4 months and underwent all the difficulties there. Now we are all in Mannar. God has spared our lives. But so many people have died. All the hard earned assets have gone with the wind.*

*Last month we went to Killinochchi. Everything was flattened. There was nothing to identify our place. Everything was overgrown like a jungle and paladainthu (in ruins). It would be verrupu (despair) to stay there. Everywhere there is only the army. They have razed the Maveerar maythanam (*Heroes (LTTE) cemetery*) to the ground and ploughed it. The place of my son's tombstone cannot be recognized. Before one day of each month I would go and cry at his tombstone. This time we were not even aware that Maveerar day had come and gone. When we lived at Killinochchi, the boys with my son would come often addressing me as ammah (mother). Now who is there for me?*

*We went to over 15 places (for assistance) to repair our houses. To whom shall we ask? When looking at what has happened to the Tamils from '77, it creates a great despair. Like the story of a illavu patha killi ( parrot that waited for its portion) our story has ended... (*with a perumuchchu*). How many people were sacrificed; hands and feet lost; houses and property destroyed; ran around as people without a country; bearing so many hardships for liberty to come again to a life of subjugation. God has also become blind. Like before I do not repeat the rosaries, do not have the heart to go to church. Only anger and sorrow comes. Before we would celebrate Christmas and New Year in a big way. This time they just came and went. Cake was not made, nor palaharam (*sweet eats*). Whom to give? In Killinochchi all our neighbours and community would come. I only go to church on Sundays because my children insist. What have we done to anybody? We have sacrificed so many people asking for freedom but only ended up without even a kachai thundu (loin cloth). We could have gone in contentment to have been killed by a shell rather than see this end. Prabhakaran has gone creating the situation where to see our house we have to get permission from the army. When thinking of everything anger wells up. I feel like burning up. There is no sleep. All the difficulties we faced keep running like a movie. Tossing and turning, there is no sleep.*

There were many stories of multiple displacements. Eventually everyone was displaced several times with decreasing periods in one place and increasing pressures from all sides, devastation and hardships. The following account by a teacher from the Mannar district maps the long convoluted journey, keeping just ahead of the direct fighting [84] up to Feb., 2009:

2.3.08- We were displaced from our village to Maddu. During this period the LTTE started to forcefully conscript our youth. Many parents, male and female youth were affected psychologically by this. Some attempted suicide by taking poison. All male and female youth were cosseted in the Madhu church while parents guarded them. Finally people were forcefully sent to other places.

3.4.08- From Madhu we were displaced to Thachinamaruthamadu. People suffered without basic facilities. Here also forced conscription continued. Some hid their children. They moved them between bunkers and jungles. Many were affected psychologically. There were many civilian casualties due to heavy shelling and aerial bombardment. Some were killed by the army's deep penetration unit claymore mines. The bus taking school children from Thachinamaruthamadu to Madhu was hit by claymore mines. Many died or were injured by this attack. Due to these attacks the free movement of people became restricted.

15.5.08 We were displaced to Periamadhu. Here some basic facilities were arranged for the people. Even here, people were subject to problems like claymore attacks, shelling and forced conscription. Many students became mentally deranged, dropping out of school and staying at home.

18.6.08 People were displaced to Ganeshapuram. NGO's and service organizations helped the people. Here also people faced continuous problems. Continuing deaths affected many mentally. There was severe shortage of water. Shelling and aerial bombardment took place.

23.7.08 We reached Anaivilunthan. As people had to leave many of their belongings on the way, they were affected psychologically. Shelling caused injuries and deaths. The successive displacements disturbed people. The education of students suffered.

11.8.08 We were displaced to a place called Puthu murripu. Here also the same problems were encountered. People were pushed to grave difficulties. Some ran out of money. Deaths increased, house to house there were funerals.

25.8.08 Displacment to Vaddakachchi. Here also all the same problems. People endured severe hardships. Forced conscription was done by beating the people and taking male and female youths. Conscripts escaped from the LTTE and returned home to be hidden there.

*7.10.08 We moved towards Tharmapuram. People had to struggle through heavy rains and flooding. Huts went underwater. Other problems cropped up. People betrayed each other to the authorities *(LTTE). *Youngsters were caught and taken away at night, at midnight. In the name of conscription, some were beaten up, some were taken away tied to poles. Male and female youth were kept in hiding. Some became frustrated and joined. There was no one to give comfort to the people, they became desolate. They were broken by shelling and gunshots. Some were taken away for border duty and other work. Some dead youth were returned to the families in coffins with their faces concealed. Everywhere there were funerals. Everyone talked about death. Youth spent their time in hiding away from their studies.*

13.1.09 People moved to Visavamadu area. Streets were crowded with moving people. Funerals were ubiquitous with smell of corpses. Bodies were buried day and night. Witnessing all this, many became mentally deranged. People were stricken by the loss of their belongings struggled without basic facilities grieved for the deaths of kith and kin. Many died unnecessarily by the shelling. Dead children to elders, lay around orphaned. Air bombings increased. Some succumbed to army firing.

30.1.09 Many people moving to Irudumadu were killed by shelling. Some left everything in a bid to survive. People were psychologically affected.

3.2.09 People moved towards Suthanthipuram. There was no drinking water, people dug holes and drank the water there. Shelling was particularly heavy. People were bewildered. On one side there was forced conscription, on the other there was continuous shelling. People lost everything, did not know where to go, what to take, what to do. Shelling was heavy even in what had been declared as the Safety Zone. Children who had lost their parents, parents who had lost their children. It was a scene that one could not look at. Bodies were buried in bunkers as it was not possible to bury them properly. Some bodies were wrapped with clothes and buried two to three feet deep. Some could not bury the dead, they simply left them and ran. Some had lost their hands, other bodies were shattered, people suffered greatly. In the hospital, there was a shortage of medicines and medical workers. People starved without adequate food. Rice and Dhal were the only food. Some broke into the stores of cooperative societies, service organizations and shops to meet their needs. There was no clean drinking water. At the same time, the LTTE shot those trying to escape into army controlled areas. Hospitals were crowded. They suffered without medical facilities. Some days were spent only in bunkers. Only for short intervals was it possible to come out. It was in this place that they faced many difficultiesPeople gave up leaving it to God's will. Nobody knew who will look after whom, who will provide comfort. We experienced suffering here that cannot be described. Many were widowed. Most people, beyond age differences, were physically and mentally affected.

**Figure 3:**
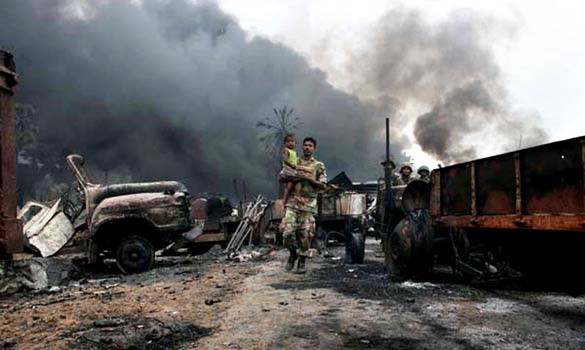
**Apocalyptic Images **[85].

The following account describes the final days at Mullivaikal in apocalyptic terms (see Figure 3[85]). There is deep emotion and resentment mixed with an earnestness to give voice to those who died there:

*Mullivaikal was where the Vanni Thamilan *(Tamil persons) *had their hair shorn and mouth gagged while nails were driven through their hands and legs like the scene at Calvary while 80 million *(world) *Tamils looked on. Out of their national interest, the ruling regime washed their hands off Tamils to kill and destroy under attractive terms such as' war for peace' and 'humanitarian action'. 300,000 Tamils were rained with shells causing rivers of blood to soak that land. Daily, I want to forget those days but the memory of the thousands who died makes me want to show the outside world happened there. That would be giving the dead souls athma shanthi *(paying respect, letting them rest in peace).

*Everyone ask us who survived that death land why we went behind them *(LTTE)*? Aren't you just ordinary civilians? From Killinochchi we were displaced to Vallipunam (Puthukudirrupu). We made a house there and stayed for a month. Shells that were falling kilometers away started falling in our yard. Seeing the dead and injured in neighboring houses our minds became disturbed and we joined those running to go where our legs took us. We changed places four to five times within a village. Wherever we went we dug bunkers like soldiers and brought together our kith and ken. We struggled to obtain food and water. It became quite clear that this war was against everything living in the Vanni. According to my reckoning there was one death for every 10 persons. Ordinary people were asked to go to Suthanthirapuram as it was made the safety zone. We found out what hell would be like there. When we saw people die in hoards with their bodies strewn, we decided to move to Iranapalai. Iranapalai was also declared a safety zone. We thought this was also a killing field. Tank shells, Kifir *(aircraft) *bombs, multi barrel missiles, kothu *(cluster) *bombs and 50 caliber gunshots targeted the people. My mind became benumbed seeing young infants to the elderly being injured and dying. Tears did not come when relations were killed. The current corpses were surrounded by future corpses cowering in bunkers. We kept hearing that people were surrendering to the army. Death was certain if we stayed. We were driven to choose. There was no food, no medicine but my family and relations were like cats that had seen fire. Because on an earlier occasion, when we had sought refuge in an army camp in Anuradhapura (birthplace of many of my relations), they had opened fire killing and injuring many. We had escaped to Killinochchi and then Jaffna where we were bombed with explosives and faeces (barrels filled with faeces) that smeared out bodies. From that time we had lost hope that as civilians we would be spared. It was imprinted in our minds that if we run in the opposite direction to the army we may somehow survive. That belief was confirmed on many occasions. But at Mullivaikal the opposite direction was the sea! When we were at Vellaimullivakal, the Tamil Nadu (in India) Chief Minister's fasting drama created some hope of a ceasefire, a restriction on the use of heavy weaponry. There was brightening in the faces of people. There was no food to eat, no water to drink, no medicine for wounds but we believed the person who represented 60 million Tamils.*

*Only that night the peak of heavy weaponry power was displayed. Artillery shells with phosphorus fell near our bunker and caught fire. Everywhere there was marana olam *(death wailing). *Even at night, with the fond hope of saving the lives of our children we ran where our legs took us. We rested on the sea shore sands. We did not believe we would be alive the next day. My aunt had been burning after being struck by a shell when we ran. When we came the next day, only her head and chest remained for us to bury in the nearby bunker. In every bunker two to three people had died. We joined those camped on the seashore. Fear of death had created a sense of comradeship with others. We were ready to share anything we had.*

**Figure 4:**
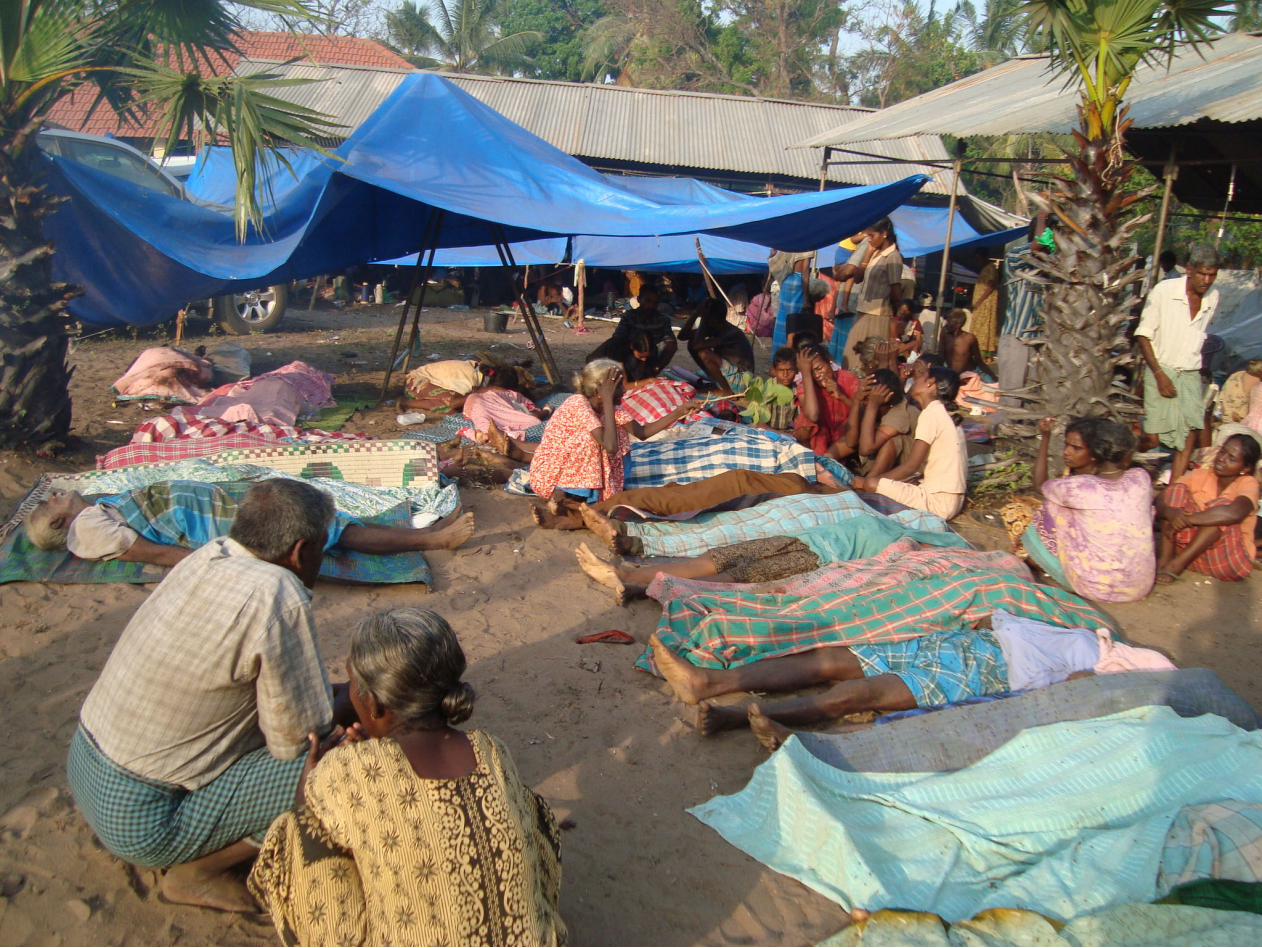
**Ubiquitous death**.

*Death was ubiquitous ( see Figure *4*). People who come out of the bunker to have a tea, fell back dead. Shells did not spare those in bunkers. My elder sister in law was injured in her head by a 'canon' attack and died due to lack of medical attention. Elders and children standing in queues for bread were slaughtered. Those who stood for gruel, to buy milk powder all went in the carnage.*

While running to Vadvahal we had to duck for cover in a palmyrah cluster by an open bunker. There were altogether ten children from my family and relations when a shell fell by the side killing our neighbor. The children were covered by sand. I was dazed thinking they had all died, when on pulling out one child, it was unharmed. When the other children were also unharmed, a belief that there was a God became strong. Finally we reached Vaddivahal. My brother was injured on the way while another was missing. The injured brother was taken away by the army and lost all his money but he survived. When we approached Vaddivahal a soldier called us females and showed it by signs. Why were men called this way? Later, why were we locked up like prisoners? There were many questions but no one to answer us.

*Many of the militants (*LTTE cadres) *who surrendered in front of our eyes were not in the ICRC register. Many said they had been shot. When will we be allowed to resettle in our own places? With the armed groups destroyed, will ordinary civilians like us be given freedom of opinion, freedom to protect our lives? Only the world can give an answer.*

This is a much more individualistic presentation elicited by a doctor from a medico-psychiatric perspective that was diagnosed as Post Traumatic Stress Disorder (PTSD):

#### Horrendous memories

**Figure 5:**
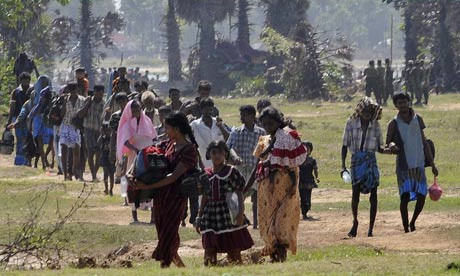
**Treacherous Pathways **[86].

*Eighteen year old Thevan was a student. His native land is Paranthan. His childhood had been happy. He had aadipaadi (played, literally sing and dance) joyfully with his companions. He was studying at high school with a goal of becoming an engineer. All his dreams were shattered by the war. Horrible shelling, artillery fire and bombings had thihil adaya (create turmoil) among appavi (innocent) folks. Thevan sought safety in many places carrying only a few belongings. Everywhere there were bunkers. On one side there was channa nerikaddi (pressure from crowds of people) while on the other side were marana olangal (wailings from death), and paddiniyal vaduhintra (starving) people. Because of the terrible war, Thevan's family entered the army controlled area on 20.4.09. They were enjoying the relief of having escaped with their lives when on the irregular, rough pathway(see Figure *5[86]*), they were unexpectedly caught up in a land mine explosion. His mother (41 years) and brother (21 years) lost their legs right there. That horrible scene happened right in front of his eyes. Thevan was also injured badly. While coming on the way, there were many dead bodies lying around. On one side there were other injured, bleeding people while on the other side there were those crying loudly for the relations who had died or been separated. Dead children's bodies were floating in the lagoon *(Fleeing people had to cross a deep lagoon (*Nandikadal*) to reach the army controlled area. Many, particularly children and elderly drowned in the crossing). *Thevan was terrified by these scenes. After great difficulty he was admitted to Vavuniya Hospital. His mental state was disturbed by memories and images of dead bodies lying around, skeletons without flesh, the scene of his mother's shattered leg due to the landmine, smell of explosives when he breathes, images of running blood and smell of blood appeared to happen repeatedly. In his sleep he hears voices, " why have you not gone to the movement? Don't you know how to fight?" He is now separated from his family. He has forebodings about his future.*

The principal of the school had referred this IDP student with educational difficulties. She was found to respond poorly to questions or activities, be withdrawn, not mixing with other students, showing fear and startling easily to small sounds. A similar situation was reported about many of the Vanni IDP students. The teacher found that the student continued to be frightened of danger to her life. Her eyes conveyed extreme fear, ever vigilant. She would startle easily, even when her name was called softly. She tended to isolate herself, not mixing with others, cried often and breathed heavily with sighs (*perumuchu*). She was apprehensive that people in uniform will abduct her. She felt that life was over, what was there for the future? Death was certain. She felt that there was a risk in speaking, that she would be put in jail. This was her story:

*From the beginning of the final war, we had been displaced 14 times. There are no words to describe what we underwent. The war continued relentlessly in the Vanni. People were constantly being displaced. Wherever we went, shells would fall and explode, injured people would struggle in pools of blood and die. Unbearable sorrow.... Father, mother and two younger sisters- we were living happily when this war took away our freedom. Not only the shells, bombings from planes, and gunfire but to escape the recruitment by the Tigers *(LTTE), *we had to be shut in bunkers and kerosene barrels. Tigers would come in vans and drag us into the van. Once inside, they would cut our hair as identification of being conscripted. After that it would be danger from both sides. My (*school) *mates who had been taken on one day would be dumped back in their homes as corpses the next day. My parents did not want this to happen to me and my sisters. As soon as people became aware that pillai piddikarar (*child catchers-LTTE) *had come signals were passed on. Immediately we would have to descend into kerosene oil barrels that were buried underground in the backyard. They would close the lid and sprinkle soil on top. There will be a small tube fitted for breathing. Waiting for about an hour or so till they leave is thihil (*nerve-racking). *We can't hear what is happening outside. Besides sweating, trembling and thinnaral (*quake*) inside, it would appear not to matter if we are caught if only we could come out of there.*

*As the shelling and airplane attacks were ahorum (*horrible*), we moved at 1 AM at night to Pokkanai. We had by then lost all our belongings. We thought that if we could just save our lives that would be enough. There were many other like us there who had put up huts. In the dry environment, the sand was hot, there was no water, no food. We had to live amidst abductions, robberies and killings without food and clothes to wear. One day, my father had gone in search of food, my mother and sisters in search of water. I was all alone. Shells continued to fall. Feeling frightened to be alone, I had come out. As I was crossing several huts, I saw that the place was surrounded by over 20 pillai piddi karar. To escape from them I started fleeing. They came chasing after me. With trepidation and desperation to escape, I hid behind huts and ran towards Puthumattalan. I got some relief only after they left. I stayed with an aunt at Puthumattalan. My parents and sisters came there by nightfall and with 150 others we decided to go into the army controlled area. The tigers came running on all four sides (t*o prevent this) *firing guns, shouting "dei, dei", hitting people with coconut stems and sticks. My heart started to pound. We didn't know what to do. We kept crying out, "help us, help us". Tigers fired wildly. Parents fought against the tigers. Some were dragged away by the tigers. The struggle went on till the next morning. The army then saved us and sent us to the Vavuniya camp. From there we were sent to Jaffna and I am still alive to be able to talk to you today.*

**Figure 6:**
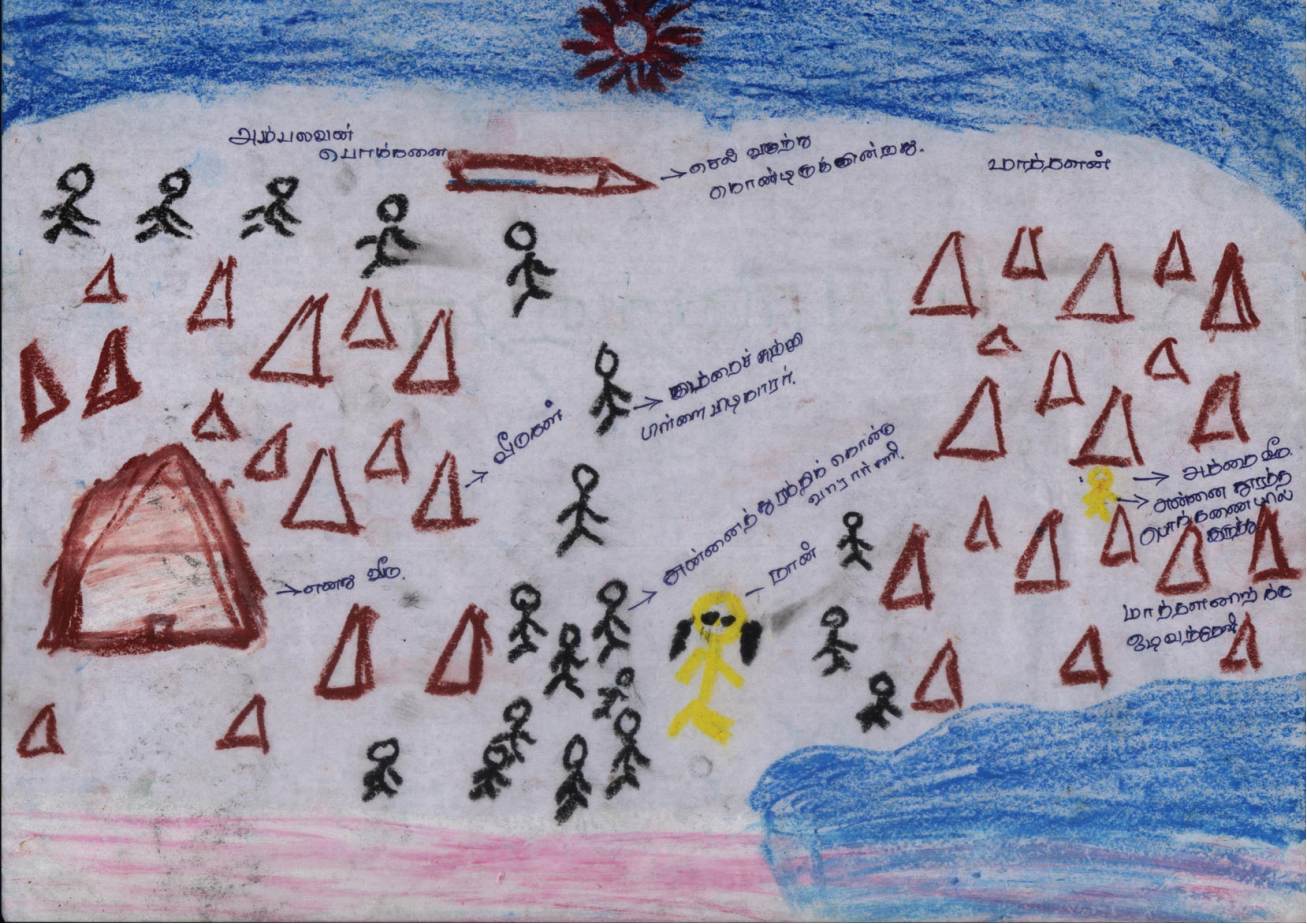
**Caught in between**. Drawing by Vanni IDP school student showing herself (in yellow) escaping from pillai p*iddikarar *(LTTE recruiters in black) amidst shelling from the Sri Lanka army through huts towards (Pudu)m*athalan *beachfront area.

When asked to draw what disturbed her most, she drew the picture (see Figure 6) showing herself (in yellow) escaping from the pillaipiddikarar (in black) amidst the continuing shelling through the huts towards (Puthu) mathalan (beach front).

The following case histories were taken from civilians recovering from serious war injuries who had been transported out with one bystander (a carer relation) to various hospitals, mainly Vavuniya. A Medicine Sans Frontiers (MSF) Nurse [87] described them: "*Wounded, shocked and distressed. After fleeing heavy fighting in the Vanni, people arriving in Vavuniya hospital need both medical care and counselling*. *People arrive here in a state of extreme anxiety and fear. They have been separated from their families and often have no news about their fate. Young children and elderly travelling with their caretakers claim they were separated at a checkpoint. The caretakers or family members who were healthy were forced to go to camps, whilst those wounded and sick had to go to the hospital. Children at the hospital are unaccompanied. They scream and call out for their mothers. Elderly people are on their own. Some people have bad wounds, some have been amputated or badly hurt by shrapnel."*

#### Look at the state I am in

*"I am 54 years old. I lived happily and comfortably with my wife and 8 children. I am very well educated, I know all three languages. We worshipped our farm work. We owned many properties and land. My children studied well, two of them even received university admissions but they *(LTTE) *didn't allow it*. (LTTE had a strict pass system, particularly for those in the recruitable age group. Some were allowed out of their area of control if someone else stood surety for them). *Why do you think I sent two of my daughters overseas to be married? Not only that, I have 11 siblings. Because of the current war situation we had been displaced 8 times, living in bunkers. One day a shell fell close by injuring my hand and leg. I was taken to a hospital and then to Trincomalee. Despite having so many relations I am now all alone. My family, estate, health and relatives - I have lost them all, if only the shell that fell had hit me, we could have died together", he said with agony*.

#### Suffering from separation

*I was 44 years old living with my husband and 4 children happily in our village. Our 4 children used to attend school while we were farmers. Due to the war situation we were displaced 11 times. To save our lives we dug bunkers wherever we went. On 8.4.09 a shell fell on top of the bunker and my daughter, son and husband were injured. While being taken to Mathalan hospital, they *(LTTE) *caught my 17 year old daughter and 15 year old son. I am here with my injured daughter. We had protected them all the way but on the way this has happened. We should have all died there. I have lost everything to become alone. "What would be the state of my husband and children?" s*he asked with grief.

#### Anguish of a 10 year old

*I had a father, mother and two siblings. My native place is Killinochchi. I am a fifth year student. Due to the current war, we were continuously displaced from 7 to 8 places. When my father and I went to the shop to buy food, a Kifir rained bombs. My father died immediately. I was lying on the street with injuries in my stomach and leg, bleeding profusely. I cried to be taken to hospital. People going on the street just looked at me. No one picked me up. Afterwards someone took me to hospital by bicycle. I came by (ICRC) ship to Padaviya and then to Vavuniya hospital with my mother. What happened comes continuously as a nightmare. I am scared. I am sad when I think of my father*.

#### What a life

I am a 47 year old male from Killinochchi. I was married with two female and one male child. A beautiful family. We were living with good facilities. Started the war. Continuous displacements. We had to live in Tharappan shacks and bunkers. Life became terrible. We had just reached Suthanthipuram, it was not even an hour had gone by when continuous shelling... , one fell on our shack. In that place two of my daughters died. Son lost both his hands and a leg. I could not even properly bury my daughters. I have brought my son here. They did not allow my wife. No news. I have searched in all the camps. Is this a life? Life has deteriorated, children are also gone, wife is also not to be found, what is the purpose of living?

#### I was not able

*I am a 47 year old married woman with two female children. Native place is Mannar District. We were doing well. Started the war. 13 times displaced. We were four siblings. I was the last. My mother was 87 years old. She was living with me. Because of this cursed war I left her with my brother. We all left together. My brother had come before. We had to cross a river on the way. My mother had left early. There were many people. I left my mother in my brother's care. My brother left my mother on the other side of the river. How much my mother would have suffered? I was not able to bring her to this side. I left her with my brother's family. I wanted to save my children and came across. I could not save her. She must have suffered so. Nobody is there to help her. Didn't she also leave because she wanted to survive? I do not know what to do...*.

#### Trembling

*I was a 48 year old male living happily with my wife and four male children. I was a fisherman. We had no shortcomings. I educated my four lions *(sons). *At this time the war started. I lost my occupation, I lost my beautiful house and property. We were displaced to three places. As we were going with what was left, there was heavy shelling. People scattered. We became separated from my four children. Suddenly to see, I was in a vehicle with my wife, one leg and hand was not there. I suffered in that state. On the way in a bus, they separated my wife and sent me alone to the Vavuniya hospital. There is no news of my children. Are they alive or not? Where is my wife? I am trembling all alone*.

#### How to go on living?

*Although I was 27 year old woman, I looked after my disabled brother, another school going brother and elderly mother who was ill, while doing handwork at home to earn a living. My father had died 7 years previously and my mother had become sickly as a result. I cared for all three, did the housework and in the time remaining made mixture *(short eats) *to sell. I was hoping that my brother would study and start working but it did not happen. Shelling and aerial bombardment did not allow us to stay in one place with any peace. We were displaced to seven placed and faced a lot of economic difficulties. At this stage in the eighth place while in a bunker with another family, I took my mother to the toilet and my brother went to fetch water when I heard a loud noise. When I looked he was lying on the ground, when I got closer his legs were missing. I ran carrying him while screaming save, "save him, save him". I kept him in the hospital there for three days. I have no news of my mother nor of my disabled brother. Now I am at the Vavuniya hospital unable to leave my 13 year old brother without legs. I do not know how to go on living*.

#### Orphan to an orphan

*I am 24 years and my wife is 24 years. It was a love marriage. We have a six month female child. Our relations have cut us off but I had a government job. We were living happily when the war started. Because this we decided to escape to Vavuniya. We left all our property and were displaced to many places. Finally in one place there was a big crowd and we were under a tree when there was a noise of a kifir bomber. All ran helter skelter. The child was in my hand. Before I realized what was happening they put us in a bus and deposited us elsewhere. I searched for my wife but could not find her. The baby was crying. Finally they brought us to a camp in Vavuniya. I do not know how to care for the baby. I am an orphan and have another orphan. I ask everyone to find my wife*.

#### Coming and going

*I was 27 years old living happily with my husband and two small children in our native village. Husband was famer with a lot of land. We were able to find enough food. The war situation made us move 3 to 4 times. We were heading for a safe place when there was heavy shelling. I do not know what happened next. When I opened my eyes I was in hospital. My mother and daughter were by my side. I was without a leg and fingers. Daughter is also injured. I learned that my husband and 2 year old daughter had passed away. I am 7 months pregnant. I do not know how I am going to give birth to this child and then bring it up. I am troubled. There are no relations here. How is our future going to be? It is forebidding*.

#### Where is peace?

*I am a 20 year old female from Urithrapuram, Killinochchi with three brothers. I have studied A/L level (year 12). I was living very happily with my mother, father and brothers who treated me as a chella pillai (favourite, spoilt child). My mother used to practice Ayurveda *(traditional) *medicine. Then the war started up again. It was mainly a bunker life. We lost our sleep and peace. We struggled to find food even for once a day. When we were displaced and in a bunker, there was sounds of many shells. We crouched in fear. Suddenly there was a loud noise close by. I lost consciousness*. (When I regained consciousness) *I found that I had lost my leg and hand. My mother was besides me to help. Then we were transferred to this hospital. I am in this handicapped state. Only my mother is here. What has happened to my father and brothers? When will we be together again? Is this my state? To think it is sorrowful *(with tears).

#### What a life?

A 60 year old woman was mumbling: *I have three married children with 10 grand children. We were displaced 14 times from our home. Food was difficult. Rice was 250, chilli powder 22, coconut 250. Rice and dhal was food. We could not take it anymore. So we tried to leave. When we were in a tarappan shack, a shell fell killing my husband, son in law, grandchildren, all together 8 people died then and there. Daughter and a grandchild were injured. So I was sent as a helper. I do not know what has happened to the rest. We have to beg even for the clothes we wear. We did not even bury the dead. Do we need a life like this? I could have died with them. Why did I come here? Have I to go on living? Those who should live have gone. What is there for me anymore...*.

#### Where is solace?

*I was a 43 year old driver from Killinochchi owning a private bus. We were well off. With 4 children we were displaced 6 times. At that time I had 5 lacks worth of goods in my vehicle. My wife and I were injured when we inside the last bunker. They *(LTTE) *had taken away my eldest daughter. I had three sons aged, 13, 11 , 9. I was injured in the head. My was injured in her chest. They brought us by ship *(ICRC) *to Padaviya. They sent my wife and children by ambulance. My wife left us *(died) *on the way to Vavuniya Hospital. I am not worried about the loss of my property or my well being. The loss of my daughter and wife is my big sorrow. I did not see my wife at her end. My children have also become alone*.

#### Why should we live?

*We have somehow survived. My 13 year old son is by my bedside with a face overwhelmed by sorrow. I suffer continuously from my leg that has been amputated above the knee due to shell injury. I cry all the time. I had tied my leg up with cloth tightly while living in bunkers for six months. We were displaced from Killinochchi three times before staying in Suthanthirapuram. We had not eaten properly for three days due to continuous shelling. Suddenly there was a lull in the shelling and my children wanted to eat some chicken. To fulfill their desire, I skinned a chicken and cooked it. After eating it we wanted to sleep. While lying down, a shell fell on our dwelling killing my wife and two children then and there. Only the two of us survived. We could also have died. What shall I do? Somebody known to us had picked me up and sent us to the Vavuniya Hospital*.

#### Separation anguish

*I am 30 years old. I was married with 4 children living happily. I never expected that our family would come to this state. At first, in January *(2009)*, four people were killed and 30 injured by shelling in our village. After seeing that we no longer wanted to stay there, we wanted to go to a place without shelling. We left only with the clothes we were wearing. But wherever we went, shelling and Kifir bombing followed us. I did not know what to do. There was rain, sun, jungles, roads, schools (*as refugee camps), all without food, water, bathing, we suffered terribly. We dug a bunker for safety and were living in a camp one day when the *sun heat was unbearable under the tharrappal. We and many others were under a tree. On my lap was my last child, others were playing when suddenly there was the sound of shell exploding. I tried to carry my child to run but couldn't. The shell fell where the children were playing. I looked thinking they all had died. A daughter was unconscious. I did not know what to do. I left the child in my arms to pick up my daughter who was unconscious and ran. She was injured in her abdomen. She needed to be treated urgently....*.

#### Remorse

*Rada is a 41 years old labourer from Killinochchi. He was married with four children. In 1990, to escape from the terrible war they had sought refuge in India. When there was relative improvement in the situation in 1996, they had returned. He was a heart patient taking treatment but was able to educate his children. They were living happily when the war broke out again. Shells started falling and exploding in their area. To safeguard his children they moved to several places with some their belongings. Their life was spent mainly in bunkers. The noise of artillery shells, firearms and bombs terrorized ordinary civilians. People ran helter-skelter seeking safety. On that 4.2.2009 when his wife (30 years) and son (7 years) had just come out of the bunker, when they were badly injured by a shell attack and lay in a pool of blood. Son died there. In the hope of at least saving the life of his wife, they took her to hospital. As the treatment was not successful, she left this world the next morning. When Rada learned of this he did not know what to do, he became benumbed. In the midst of heavy shelling they could not carry out the burial of his wife and son properly. Returning to their shelter with his remaining three children, Rada could not control his mind. He found all his belongings had been destroyed. In this terrible state, on an impulse he tried to consume poison and also give to his children. The children cried loudly. His 16 year old son thwarted the suicidal attempt. Then Rada decided to save the lives of at least his remaining children, joined a crowd of escaping refugees on 7.2.2009 and reached Vavuniya. They are now at the Gamini school camp. Having lost two lives to the horrible war, those thoughts came recurring daily to Rada. He was found to have lack of appetite, sleep, crying without realizing it, unable to socialize with others, suicidal ideation, not knowing what to do next, headache, numbing of the head, worry about the future of his three children and a deep depression. He felt remorse about not doing the funeral rites of his wife and son. He is without the support or help of his relations*.

#### Guilt

Fifty one year old Siva was born in Killinochchi and worked as farmer. He was married with four children. The eldest was married with a child and his daughter was a school teacher. They had escaped from shell attacks to live in bunkers at Sudanthirapuram. A shell fell there killing the eldest son and daughter. A son was injured in his chest and leg while his wife escaped with minor injuries. His daughter in law and child are in a refugee camp in Vavuniya His injured son is at Mannar hospital while his wife is in another camp. He is with his daughter at the Pampaimadu 7^th ^mile camp unable to contact his siblings or relations and without contact with his injured son, daughter in law and child and wife. He is severely depressed with continuous crying, loss of appetite, lack of sleep, repeated memories of what happened in the Vanni, poor self-care and headache. On counseling, he cried, revealing that images of his two children dying in front of him and their leaving their bodies in the bunker without even carrying out their funeral rites keeps recurring in his mind preventing his sleep. As it was now one month since the event, He felt especially guilty that he was not even able to arrange the customary 45 day remembrance ceremony for them

#### Widowed and pregnant

*24 year old Mrs. Kavitha was 8 months pregnant and mother of 4 year old son. Her husband was an ordinary labourer. They had been married 5 years and was going in a happy direction when they had to flee for their safety when the dreadful war broke out. Everywhere there were the sights and sounds of shells attacks and reverberating sounds of gunfire. In many places there were the kifir bombings. People experienced allola kallola *(pandemonium)*.They ran seeking shelter. Their daily lives were spent in bunkers. Everywhere there was marana olangal (*death wailing*) with deaths from very young children to the elderly falling victims to the awful war. It was in these circumstances that Kavitha floundered having lost all her belongings, separated from relations. Facing great difficulty her family tried to reach the army controlled area when her husband was shot by the armed group *(LTTE). *In that place there were many people with fatal injuries lying in pools of blood. When Kavitha looked at her husband he was in dead posture. To save her child, she left her husband's body and joined other people to attain the army controlled region. She is currently living in a IDP welfare camp with her four year old child. She is without contact of her relations. She was in deep thought about her upcoming delivery period and future life. She helplessly asked, "Who will look after my four year old when I give birth?" Kavitha was found in a disturbed mental state with loss of appetite, lack of sleep, recurring thoughts relating to her husband being supported by her four year old in the welfare camp*.

#### Hopelessness

*Somu was a 30 year old male married with a 18 months old son. On that day, he had left his wife and child in a safe place to go and bring his mother and sisters. Youngest sister was a final year university student while the other sisters were married. When they had started to leave with their belongings, the army had seen them and started firing. His mother, sister and one baby died then and there. His youngest sister had fled. He had run after her fearing that she would be caught by the army and raped. Bullets pierced his neck and chest. The next day he regained consciousness hearing the voice of soldiers who had come there. They kicked him asking, "where are the others?". He begged them, "you have killed the others, kill me also." He was in a state of extreme distress and frustration at Vavuniya Hospital without knowing what had happened to his sister and without information about his wife and child. It was found that his legs and body would not function. He was unable to lift his neck due to the injury in the neck. He had repeated thoughts about his sister and what had happened that day. He had lost all hope about his state*.

#### Shattered dreams

*My name is Ravi, a 15 year old born and bred in Killinochchi with two sisters, mother and father who was a car mechanic. Being a keen student, I had succeeded in the fifth year scholarship and was continuing my education at the Killinochchi Mahavidiyalayam *(high school). *When the war broke out again in 2006, the Tigers made many attempts to conscript me under their ' veedukoruvar *(one person for each house)' *policy. While I was returning from school they tried to forcefully abduct me in their vehicle. Somehow I escaped through by-lanes leaving my bicycle behind to reach home. This happened in January, 2008. After that I stopped going to school. My parents also stopped my sisters from attending school. I could not study. I could not come out of my home. My life was frustrating. In the evenings, I used to play football for an hour at the Thirunagar grounds. That was blocked. People found me full of anger and despair. I would often get into fights with my father. I would say we should have gone to Vavuniya during the peace period. How long not to go to school, tuition and the grounds? If these are not to be, I will go and join them (*LTTE). *My parents were very concerned about me. They were unable to do anything*.

*Under our Margosa tree, I had dug a bunker. As soon as I heard the sound of Kifir (*planes) *I would be the first into the bunker. Then would come my sisters, then mother and finally, father. Every day we would be going inside at least five times. As soon as I heard the sound of Kifir, without realizing it I would develop palpitations and find it difficult to breathe. I would feel agitated. When it dived (*high pitched sound of diving) *to bomb, I would Veerudu (*piercingly*) scream. Its (*Kifir) *sound was that terrorizing*.

*We had some relief at night. At the beginning we had electricity for two hours. I studied with that help. I would watch TV for a short while. There was only the Nidharshanam *(LTTE TV programme) *service. They only showed only dramas and pictures (*movies*) related to war. Daily they would show the ghastly pictures of peoples killed by shells and aerial bombing. My body would tremble when I looked at them. Feelings of antagonism, frustration and hatred towards the government forces would arise in me without my realization*.

*As the fighting got closer and closer, we first moved from Thirunagar to Tharmapuram. We put up a tent in a small plot and stayed there. We had no toilets or clean water. In the monsoon rains our tent was blown away. We had to live in two feet deep water for two days. With all that, I somehow appeared for the "O" level (*year 10 GCE national exam) *held last December *(2008) *at the Tharmapuram school. I still hoped for good results to study "A" level science and become a doctor*.

*When the fighting passed Paranthan and came towards Tharmapuram, we moved to Visuvamadu. We put up a tent on land belonging to my father's friend and lived there. February 10^th ^*(2009) *there was heavy shelling. The army was advancing towards Visuvamadu. As our bunker had filled with water we could not stay there. At about 1 PM when we had come out the bunker this horrible incident occurred. A shell that came from nowhere landed on our tent and exploded. Everywhere there was the sound of crying. I lay in a pool of blood, moaning. I could not get up and walk. On my side was my sister without any sound. Only my father was uninjured. When he picked me up crying loudly with oppari (*weiling), *my two arms were not in my control. I could not move them. I was able to move only my right thumb. Amidst all these difficulties, I was admitted to Puthukudirrupu hospital and underwent surgery. When I opened my eyes the next day my world was darkened. My two sisters who I had uyiruku uyirai nesitha (loved as my own life) had died in the shelling. My father had buried them in that bunker itself. He had brought me and my mother to hospital. My two arms were amputated and my other injures were dressed. On my side lay my mother who had had her right leg amputated below the knee. In this misery, we were taken by the Red Cross *(ICRC) *ship to Trincomalee Hospital. After one week there, we were sent to Mannar Hospital*.

Now my whole life has become full of gloom. I still have the dream of becoming a doctor. "Can I study with prosthetic arms, doctor? Please help me."

#### Helplessness

*50 year old Vani was a shopkeeper from Tharmpuram with three children. The eldest daughter was married with two daughters. The second son had been forcefully taken away by the Iyakam *(movement- LTTE) *a year ago. When the fighting became severe in January, they loaded their belongings in a landmaster and with other village folk were displaced from place to place. The 13*^*th *^*displacement was to what had been declared a safety zone, Iranaipalam where they stayed for 10 days. People had made tents to live in. As shells had started falling on that day, they loaded their goods onto the landmaster and decided to move on. But her 8 year old granddaughter insisted on having fried fish, they delayed to cook a meal. Varatharany and her daughter busied themselves in cooking while her husband, son and grandchildren were sleeping in the tent. Her Son-in-law had gone to the market*.

*That day, 21/2/2009, morning at 11:45 AM a shell fired by the army completely buried their families happiness in a deep hole and made them nirkathy (*helpless). *The shell not only landed on the landmaster burning it, but also pali eduthiduthu (*killed) *her husband, son and two grandchildren. Varatharany and her daughter were injured. On hearing her daughter wailing "my children are dead", she had gone slowly when she saw her husband lying dead, her grandchildren with their bodies thundikapadu *(severed) *and her son injured in his head, arms and legs struggling to live. She collapsed there. On hearing her distraught daughter who despite bleeding profusely had run over and was trying to pick up her kuttuyir (*barely alive, process of dying*) children while lamenting loudly, bystanders had come and taken them to hospital. The son-in-law had come later and buried the dead with the help of others there*.

*Both of them were treated for two days at Trincomalee hospital, then sent to Vavuniya hospital and currently at the Saiva Prihasa School refugee camp. For the last one month, Varatharani and her daughter have been continuously crying with constant memories of their children and re-experiencing what happened. They are disturbed by suicidal thoughts, fatigue, insomnia, and guilt feelings. Although they say that her son-in-law is their comfort, when he is alone he laments loudly saying, "We have lost our relations and our belongings. There is no point in having come here. I am useless" *(he had survival guilt, of not having been there to help his family when the deaths happened).

### Interviews

**Figure 7:**
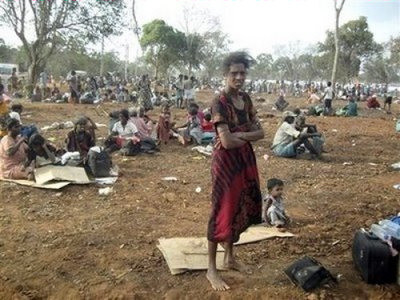
**Utter Devastation **[88].

Key-informant, family and extended family interviews and focus group discussions regarding family and community level changes indicated mostly negative but also positive developments (see Table 1). Generally there was consensus that family and community life had suffered due to deaths, separations and deprivations. Relationships, trust, cohesion, beliefs and ethical values had declined, some said deteriorated, destroyed. Instead there was an increase in misunderstandings, conflict, selfishness, suspicions, anger, bitterness, *virakthy *(loss of interest), *veruppu *( state of detestation), *soham *(sorrow), alcoholism and sexual laxity. The problems associated with the increase in alcoholism and sexual laxity has been raised consistently by health workers in the camps. Expression of survival guilt was common, particularly after the experiences in the internment camps. After losing so many of their relations or not knowing their whereabouts, many said they could rather have died in the shelling. Outward blame for what happened was common, some blamed the government; others India (*vaddakathiyar*- northerners) and some the LTTE. There was anger and feelings of betrayal by the LTTE. In the immediate aftermath, many were distraught, dazed and disoriented; there were strong feelings of disillusionment, bewilderment, disbelief, bitterness and utter devastation (see Figure 7[88]). Some said it was the fate of the Tamils (*thalaivithiy*), 'of having been born Tamil in this country'. Most felt that there had been a decline in religious beliefs and practices, loss of faith and fervour. One widow described how she and her children had left her husband who had been shattered by a shell but still alive and struggling on the road to escape themselves. She is haunted by this memory and blamed God for creating the terrible situation (*pallapona kadavul*). But others mentioned that it was only religion and faith in god that had sustained them when everything else failed. Their only trust was that God would find a way out for them. Some mentioned an increase in new relationships; mutual help and co-operation; a sense of unity, comaradeship and togetherness by being thrown together against adversity which was marked during the last days of the 'final war' and thereafter for a short period but had progressively decreased. A common observation was that people had become dependent on handouts, used to welfare and decline in efforts to work and earn. People had betrayed (*kaddikodduthu*) others for benefits and privilege from the army and authorities. But, now with the resettlement process, motivation to rebuild their lives and livelihood was strong. There was a sense with some exceptions (those who had suffered and lost most) that their situation was improving and there was hope for the future compared to how it was one year ago. There were some positive stories of resilience and post-traumatic growth. A senior government officer and writer said that they had gone through great hardship (*peravalam*), but that they now only needed to get back their infrastructure, resources, occupational opportunities and jobs to rebuild and restart their lives. He denied any ill effects like poor sleep, bad dreams or loss of motivation. He appeared in good health and committed to contribute to the resettlement and rehabilitation process. A recent (2010) observation of what is happening in the Vanni echoes this positive hope, "*the spirit of the Tamils in the north has not been extinguished by the long years of war and its brutal end. All indications point that the Tamils will rise again to play a meaningful role in Sri Lanka and prosper. The spirit that is manifesting itself in numerous ways all over the north, despite the all too obvious adversities and disadvantages, is definitely a harbinger of a bright future for the Tamils and Sri Lanka. If they are helped and guided, they will advance faster. If not, they will yet become a great people, though at a slower pace. The Tamils will emerge from their prolonged tragedy and the associated misery, despite their politicians, bureaucrats and malcontents- both within and the Diaspora, to become what they deserve to be in the land of their birth and life. I may not live to see this happen, but will die convinced, it will happen. Tamils are not a species, destined for extinction in Sri Lanka, as many, including me had feared six months back. They are proving that they have what it takes to rebound from adversity and hurdles, to survive and prosper*" [89]. A young doctor who had served through the last days of the fighting said that he had seen terrible injuries and deaths, struggled through the heavy shelling and firing at the different hospitals, working without rest. At one stage he had lost all fear and was able to continue working amidst all the chaos. He was ready to do anything. He was now seen to be extraordinarily dedicated, motivated, a tireless worker and administrator appreciated by all. An expatriate medic also described the last days of fighting as harrowing but "*After looking at the people dying and dead bodies everywhere, it is like nothing threatens me anymore, it is like I have had the hard time in my life and I think I am prepared to take up whatever happens in life now. I'm not that old Vany that sits down and cries for little things. I'm stronger now after going through and seeing all that problem. My mind is clear now"*[90]*.*

**Table 1:** Collective Trauma-Theoretical model

Disasters	Causal Conditions	X	Ecological context	→	Intervening Conditions	→	Coping strategies	→	Consequences
Man Made- *War*	Displacements		Social chaos, uprooting		Insecurity		Silence, Withdrawal, isolation, Benumbing		Dependency, learned helplessness, passivity

Natural- *Tsunami*	Separations		Breakdown of social structures and institutions		Terror		Suspicion		Distrust, mistrust, paranoia

	Massive destruction		Unemployment, poverty		Impunity, social injustice		'Fight, flight or freeze', Survival, escape, Suicide		Despair, disbelief, amotivation, hopelessness

	Multiple deaths		Starvation, hunger, malnutrition		Breakdown of law and order				Loss of communality, decrease in social cohesion, tearing of social fabric, Loss of social capital

	Injuries		Lack of medical care, diseases, epidemics		Inequity, discrimination		Cultural practices, rituals		Adaptive changes in memory, reframing, meaning, realism

	Cultural & social bereavements		'Repressive ecology', violence, torture, abductions, detentions, disappearances, extrajudicial killings,		Helplessness, hopelessness		Adaptation, facing the challenge, problem solving		Resilience, forbearance, new networks, friendship, relationships, hope

	Losses				Rumours, disinformation				Regeneration, development, progress

## Discussion

There are several themes emerging from accounts of what happened. A striking theme to emerge from the narratives is the collective nature of the trauma. All the stories describe what happened to them as a family or in some cases, to the community. Western research and conceptualizations have been primarily individualistic in orientation [91]. The fields of social theory, modern medicine, research and academic activities in general are dominated and monopolized by the western individual oriented paradigm. However, in collectivistic, co-operative societies [78,92], there is a need to go beyond the individual to the family, group, village, community and social levels to more fully understand what is going on in the individual, whether it be his/her development, behaviour, perceptions, consciousness, experiences or responses to stress and trauma as well as design effective interventions to help in the recovery and rehabilitation of not only the affected individuals but also their families and community[93-96]. For when the family and/or community regained their equilibrium and healthy functioning, there was often improvement in the individual member's wellbeing as well. Family and social support, networks, relationships and the sense of community appears to be a vital protective factor for the individual and their families and important in their recovery. This broader, holistic perspective becomes paramount in non-western, 'collectivist' or co-operative cultures which have traditionally been family and community oriented, the individual tending to become submerged in the wider concerns[29,78,92,97]. The family and community are part of the self, their identity and consciousness. The demarcation or boundary between the individual self and the outside becomes blurred. The well being of the individual member is experienced as the wellbeing of the family and community. For example, Tamil families, due to close and strong bonds and cohesiveness in nuclear and extended families, tend to function and respond to external threat or trauma as a unit rather than as individual members. They share the experience and perceive the event in a particular way. During times of traumatic experiences, the family will come together with solidarity to face the threat as a unit and provide mutual support and protection. In time the family will act to define and interpret the traumatic event, give it structure and assign a common meaning, as well as evolve strategies to cope with the stress. Thus it may be more appropriate to talk in terms of family dynamics rather than of individual personalities. There may be some individual variation in manifestation, depending on their responsibilities and roles within the family and personal characteristics, while some may become the scapegoat in the family dynamics that ensues (see family case histories[42]). Similarly, in the Tamil communities, the village and its people, way of life and environment provided organic roots, a sustaining support system, nourishing environment and network of relationships. The village traditions, structures and institutions were the foundations and framework for their daily life. In the Tamil culture, a person's identity was defined to a large extent by their village or *uur *of origin [98]. Their *uur *more or less placed the person in a particular socio-cultural matrix. However, within communities, there may be exclusion, ostracization, powerlessness, marginalization, silencing and stigmatization of some members, families, castes or groups while others seek prower and privilege. The social institution of a traditional *uur *has also undergone tremendous breakdown with the chronic war and displacements as well as modernity.

It is becoming clear that social and cultural values, beliefs and perceptions will shape how traumatic events impact on the individual, family and community and the way they respond [99,100]. The meaning attributed to the event(s), the historical and social context, as well as community coping strategies determines the impact and consequences of trauma (Table 1). The narratives clearly show the impact of the war on the family and community. The exclusively individual perspective characteristic of western narratives is completely lacking here. There are hardly any spontaneous complains of individual symptoms or suffering. Even where a person talks of his or her personal agony, it is framed in general terms, reflecting what happened to the family or community. Undoubtedly, individual symptoms, how the trauma had affected each member can be elicited with direct questioning [42] as in the PTSD example above. But in this study, the narrative was allowed to flow naturally. The story usually began with the family described metaphorically as living happily in their village. It is significant that the happiness or wellbeing is perceived and experienced in terms of the family and community. There is a dynamic equilibrium, harmony within the family and community, a network of mutually supportive relationships and responsibilities, ritualistic practices and living patterns that they have managed to establish despite the harsh socio-economic and political conditions. Their feeling of strength and value is more in those bonds and relationships not so much in the material and external circumstances. The war is seen as an imposition coming from outside, disturbing this atmosphere of contentment where the family and community was progressing, getting on with life. The war is invariably described in very negative terms, *por arrakan *( war devil), *kodum (*horrible), *per avalam *(great calamity). As the narrative unfolds, it is the family that is the focus. The shelling and fighting approaching their homes, their village, impels them to start the displacement process. They describe how they leave as a family, as a community- whole villages, taking whatever they can load onto vehicles, hoping to return in a day to two. The dispersion begins. Initially they are separated from the supportive context of their community, extended family and village. How the new conditions start affecting the family, how each member suffers, the deaths and injuries, how the separations form those who are injured, having to bury the dead without the customary rites, the guilt of leaving relations behind, and the strong yearning to know what happened to other members. The impact of the disaster is felt acutely within this living fabric of the family and community: the utter hopelessness, helplessness and devastation when the fabric is torn.

In these circumstances the best approach to restore the psychosocial and mental health of the Vanni IDP's according to mental health professionals working in the internment camps as well as clearly recommended in the Interagency Standing Committee (IASC) guidelines for mental health and psychosocial support in emergency settings[101] would have been to re-unify the family, give information on their fate and whereabouts. The second best strategy would have been to release them to find their own way and reunite with their families and community. However, the state strictly resisted these well meant efforts. If one is to extrapolate from the decisions and restrictions being placed by the authorities, discern the pattern behind the policies from past analysis [61] and experience to understand the mindset [51], the operating paradigm, it would appear that the state still fears a regrouping of the destroyed LTTE, but more harbours a deep paranoia based on ethnocentric perceptions of the 'other' [41] to prevent any future minority mobilization. There was only limited psychosocial support, while counselling or cultural healing practices either in the camps or resettlements was severely restricted [2,102,103]. In the post-conflict, military and politically sensitive situation, dealing with the mental health and psychosocial needs of the Vanni IDP's was a difficult and challenging task. A small team of mental health professionals and few NGO's with limited resources attempted to address the immediate and urgent needs. The priority was given to severe mental illness, particularly psychosis, which needed medication and intensive care. Some chronic patients had relapsed or developed exacerbation in their symptoms when they had run out of drugs or simply stopped taking them. A large number had been displaced from long care institutions in the Vanni, *Vetti mannai and Santhosam*, which were caring for over 100 chronic patients from all over the island. Some had developed psychotic illness anew. Clinics were held in the camps and Vavuniya hospital while in ward treatment was available at the General Hospital. Similar secondary and tertiary care was available in Jaffna and Mannar. However when it came to addressing the psychosocial needs, access was limited. Ingenious strategies had to be adapted to gain access and provide support despite the military presence. A group of community level workers, Community Support Officers (CSO's), who had been trained after the tsunami under a Ministry of Health/WHO programme [104,105] to work with the affected population in the Vanni were among the IDP's in the camps at Vavuniya. They were again mobilized by a Ministry of Health/WHO programme to work among the IDP's. Some other psychosocial NGO's did yeomen service under trying circumstances. Nevertheless, consistent and systemic long term programmes were not allowed. The Mental Health Consultative Forum for the Northern Province consisting of mental health professionals and health administrators from the health department was formed in November, 2009 to deal with the Mental Health needs in the resettlement process of the IDP's. The Forum has formulated a plan to mobilize those already trained and skilled in community level mental health to form a network of psychosocial support at the periphery (Divisional (AGA) or District Levels (GA)). Other community level and governmental workers can be trained. Training of grass root community level workers in basic mental health knowledge and skills is the easiest way of reaching a large population. They in turn would increase general awareness and disseminate the knowledge as well as do preventive and promotional work. The majority of minor mental health problems could be managed by community level workers and others referred to the appropriate level. The main effort of community level workers would be directed towards strengthening and uniting families; rebuilding and regenerating community structures and institutions; encouraging leaders; facilitating self-support groups; village and traditional resources; using creative arts; cultural, ritualistic practices; as well as linking up with other service sectors like education, social service, local and regional government. However, the state does not recognize the concept of psychosocial needs or support. For example, knowledge that apart from other physical and socio-economic needs, it will take considerable time and psychosocial support for the people to get over their trauma is not accepted. The Vanni IDP's will have to be given an opportunity to mourn for the dead, grieve for the losses and practice the cultural rituals for collective consolation. What happened cannot simply be erased from collective memory. If proper healing and psychosocial restitution is not done properly or they are pushed into activities too quickly, they may not benefit fully from the resettlement, rehabilitation and development efforts. They will lack the motivation and well being to participate fully in their recovery and rebuild their homes, lives and the region. Nevertheless, in the long term, one would expect the Tamil community to eventually recover despite the malfeasance [89]. Although it is a much more complex and chronic sociopolitical situation in Lanka, the community's resilience that lies in its strong identity, culture, social and spiritual practices will help heal the wounds as happened naturally, despite all the shortcomings and neglect, after hurricane Katrina in New Orleans [106].

A broader and long term psychosocial intervention for collective catharsis and a healing of memories for traumatized families and community would be an acknowledgement of what happened. Apparently the state did not want the stories to get out for fear of prosecution for war crimes that was being put forward by some members of the local and International community [2]. It continued to insist that '*not a single drop of civilian blood had been shed*' and the '*biggest humanitarian rescue mission in history*' had been executed [107]. The politics of memory and history writing are linked to power. Those with the power to impose their version can change memory traces and perceptions of what happened. The LTTE managed to enforce their account of the 1995 exodus in the memory and imagination of Tamils as resulting from state action when they in fact engineered a movement of over 400,000 people from Jaffna [53]. The Jaffna exodus, many of whom ended up in the Vanni, and its context had many similarities to what happened later in the Vanni except that there wasn't such large scale civilian deaths and injuries. The LTTE then chose to withdraw into the Vanni jungles rather than make a last stand in Jaffna with civilians, avoiding a similar humanitarian disaster[54].

Around thirty thousand civilians appear to have been killed and scores more injured in a short period with large scale, repeated displacements, shortages and neglect of basic needs such as food, shelter and medical care. Allegations of war crimes, and crimes against humanity have been raised at the highest levels calling for investigations and persecution by world bodies [1-3,6-8,69,70,108]. There have also been heavy casualties among the army. According to reliable reports around 5000 soldiers died while many times more were injured in the final push [109,110]. Perhaps 7,000 LTTE militants died or were executed in 2009 alone. Many were raw conscripts pressed gang into battle to became cannon fodder. From past experience with such battles and casualty figures, a conservative estimate for the whole Vanni battle may be well over 10,000 killed for each side. The story of ordinary soldiers and militants also needs to be told; their sacrifices, suffering and agony recognized; accepted for healing of their memories; and ultimately, for national reconciliation. It becomes abundantly clear that both the Sri Lankan state and the LTTE are responsible for serious human rights violations on a large scale. Though indictments or establishing moral responsibility may not be realistic in the current international, regional and local political context; at least, reinstituting a belief in social justice would be an important psychosocial intervention for communal harmony and wellbeing as well as the future of the country.

**Figure 8:**
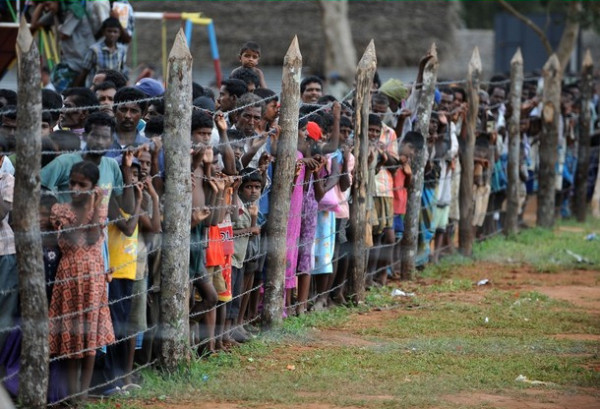
**Internment Camps **[111].

However, another interesting theme that emerges from the narratives is the contest for the loyalty or obedience, the so called 'hearts and minds' exercise, that operated to a large extent at the unconscious level. Evidently the Vanni civilians had some allegiance to the LTTE up to the beginning of the last phase of fighting in 2006. Many believed in the LTTE version of the 'freedom struggle' and had chosen to go to the Vanni, for example during the 1995 exodus from Jaffna [53], and stayed on despite the hardships and shortages. There had been considerable compulsion in making this 'choice' applied by the LTTE which also had a strict pass system preventing people leaving their area of control. Nevertheless, the LTTE and their sympathizers perceived the Vanni people as their loyal subjects with subtle gradation of animosity to Tamils living outside. A view shared by the Sinhala State as shown by their treatment of the Vanni IDP's after the conflict. Their forced internment in barbed wire camps was obviously a collective punishment for their 'crime' of staying in the Vanni with the LTTE (see Figure 8[111]). Those coming later in the battle were considered 'more loyal', particularly those who 'stayed' till the last. They were treated more harshly and punitively with far more restrictions in different zonal camps[112]. After the 1995 exodus, those who had stayed behind in Jaffna were issued 'Army' Identity Cards with differences entitling privileged status. The LTTE and people of the Vanni also considered them as somehow having betrayed the cause and enjoying special luxuries. As the fighting evolved with the Vanni civilians facing increasing harsher conditions of ubiquitous death, injuries, conscription, multiple displacements and shortages of food and other basic necessaries; this loyalty could be seen to gradually change. Under the totalitarian fascist control of the LTTE, any kind of dissent or counter views had been eliminated. People had adapted to this state of affairs despite embargos, restrictions and attacks by the state showing considerable resilience. They were content in many ways as expressed in the narratives metaphorically as being 'happy'.

The narratives speak of the beginning of the last phase of the war in particularly apocalyptical terms. But the criticism and antagonism to the actions of the LTTE starts creeping into the narratives much later. Many show a strong reluctance to name the LTTE directly, always using indirect terms. Some completely leave the actions and atrocities being done by the LTTE out of their accounts [80,90]. Apart from their more overt repression and terror, the LTTE had succeeded in establishing this kind of collective internal censor that prevented people seeing their negative side but more insidious, thinking or speaking about it. Partly this was due to terror and a survival strategy, but it was also a result of the discriminatory policies of the state and the harsh actions of its security forces. But as the price for this loyalty mounted with increasing death, injuries and conscription, the tide turned and people became more conscious of the real nature of the LTTE. It would appear that this was a deliberate 'psyops' military strategy of the state to drive a wedge between the civilians and the LTTE, as they increased the harsh conditions: shelling causing death and injuries even in hospitals and state declared safety zones, restrictions on food, medicine and other basic items[113]. The counter insurgency (CI) strategy appeared to have worked with people becoming more overt in their resistance to the LTTE, more open in criticism and defiance, at times breaking out into direct clashes [2,5]; finally escaping over to army control. Some narratives expressed gratitude to the state forces for having saved them from the LTTE. The state has continued to use this CI strategy to completely wean the Vanni people from the LTTE after the conflict by interning them in IDP camps with callous restrictions. They have sought to impose their version of the discourse in contrast to the ideas of liberation, Tamil homeland and separation. However, instead of using the historic opportunity for national reconciliation, the repressive ethnocentric approach without dealing with the underlying grievances in the long term will only alienate the minorities once again. Apart from the political implications, the contest of the different discourses at stake and the need of the Vanni IDP trauma for healing; if not social justice, the whole national reconciliation process at least needs some acknowledgement of what happened. If there is no healing of memories, merely a repression, the untreated collective trauma could well turn into resentment and rekindle cycles of violence once again.

## Conclusions

The psychosocial and mental health consequences of massive trauma to individuals, families and communities can be profound. The interventions for recovery and regeneration should be holistic, integrated and multisectorial (Table 1). However, the underlying political context and struggle for control, power, discourse and obedience complicates what is allowed and can be done.

The following poem [114], *Shady Trees*, by a child soldier yearns for the solace from a caring, nurturing elder, community, society that has been laid waste by war:

*In our livesThere is no peaceIn our treesThere is no lifeThe dead ones become firewoodThe green ones give shade*

*The onlooker...You tell usWhich tree are weWill you ease our worries?Will you wipe our tears?We are waitingFor the shady trees...*

## Competing interests

Tamil medical officer working in northern Sri Lanka

## Authors' contributions

DJS was responsible for the study and writing of the manuscript.
